# Regional transcriptome analysis of AMPA and GABA_A_ receptor subunit expression generates E/I signatures of the human brain

**DOI:** 10.1038/s41598-020-68165-1

**Published:** 2020-07-09

**Authors:** Kevin Shen, Tommaso Zeppillo, Agenor Limon

**Affiliations:** 1 Gladstone Institute of Neurological Disease, University of California, San Francisco, USA; 2 Department of Life Sciences, B.R.A.I.N., Centre for Neuroscience, University of Trieste, Trieste, Italy; 3 Department of Neurology, Mitchell Center for Neurodegenerative Diseases, School of Medicine, University of Texas Medical Branch, 10.138B. Medical Research Building, Galveston, TX 77555 USA

**Keywords:** Microarray analysis, Ion channels in the nervous system

## Abstract

Theoretical and experimental work has demonstrated that excitatory (E) and inhibitory (I) currents within cortical circuits stabilize to a balanced state. This E/I balance, observed from single neuron to network levels, has a fundamental role in proper brain function and its impairment has been linked to numerous brain disorders. Over recent years, large amount of microarray and RNA-Sequencing datasets have been collected, however few studies have made use of these resources for exploring the balance of global gene expression levels between excitatory AMPA receptors (AMPARs) and inhibitory GABA_A_ receptors. Here, we analyzed the relative relationships between these receptors to generate a basic transcriptional marker of E/I ratio. Using publicly available data from the Allen Brain Institute, we generated whole brain and regional signatures of AMPAR subunit gene expression in healthy human brains as well as the transcriptional E/I (*t*E/I) ratio. Then we refined the *t*E/I ratio to cell-type signatures in the mouse brain using data from the Gene Expression Omnibus. Lastly, we applied our workflow to developmental data from the Allen Brain Institute and revealed spatially and temporally controlled changes in the *t*E/I ratio during the embryonic and early postnatal stages that ultimately lead to the *t*E/I balance in adults.

## Introduction

Theoretical and experimental work has shown that fast excitatory (E) and inhibitory (I) currents within cortical circuits stabilize rapidly to a balanced state^1–6^. This E/I balance, observed from single neuron to recurrent connected network levels, produces robust neuronal learning capabilities while minimizing disturbance by output noise^7^. The balance’s fundamental role in proper brain function has been demonstrated via pharmacological blocking of cortical inhibition which led to cortical epileptic activity and loss in stimuli features decoding^8,9^. Therefore, disturbances to this balance have been linked to numerous psychiatric, neurodevelopmental, and neurodegenerative disorders including schizophrenia, autism, and Alzheimer’s disease^10–14^. At the synaptic level, the electrophysiological E/I (*e*E/I) balance is driven mainly by the concerted activity of excitatory α-amino-3-hydroxy-5-methyl-4-isoxazolepropionic acid receptors (AMPARs) and inhibitory γ-aminobutyric acid type A receptors (GABA_A_Rs)^15^; however, it remains unknown to what extent the transcription levels of each receptor are also balanced across brain regions and developmental stages. This information would prove highly important and useful since direct measurement of the activity of neurotransmitter receptors at the single neuron level in healthy humans is not currently possible with available technology. Rather, differential gene expression of postmortem tissue is the most widely used approach in making inferences of neurotransmitter function in physiological and diseased states.

Our previous work demonstrated a bioinformatics approach to analyzing the organizational layout of GABA_A_Rs in the human brain. This approach proposed the most abundant isoforms that followed a preferential pentameric arrangement in the human brain and showed that gene co-expression patterns of GABA_A_R subunits are highly stereotypical within structures characterized by recurrent cytoarchitecture^16^. Here, we shift our focus to glutamatergic AMPARs. These receptors mediate the post-synaptic depolarization necessary for fast excitatory transmission and participate in the long-term potentiation and depression necessary for appropriate function of cortical circuits^17^. Unlike the highly diverse and heterogeneous GABA_A_Rs^18^, AMPARs are constructed as dimers of dimers^19,20^ from a pool of only four subunits (GluA1–GluA4)^21^ transcribed from four genes (GRIA1–GRIA4). This construction has been suggested to be highly dependent on afferent type^22,23^ and can change with synaptic activity^24,25^. Stoichiometric alterations and single subunit defects of AMPAR tetramers have been correlated with many brain disorders^26^. For instance, reductions in GluA2 and GluA3 subunits were found in the hippocampus of patients suffering from mild to severe forms of Alzheimer’s disease^26^, and alterations in GluA1-4 expression level have been observed in different human brain structures of people diagnosed with schizophrenia^27–29^. Therefore, understanding the organizational layout of these receptors should prove useful in determining pathological remodeling of AMPARs, and consequently alterations to the E/I balance.

**Figure 1:**
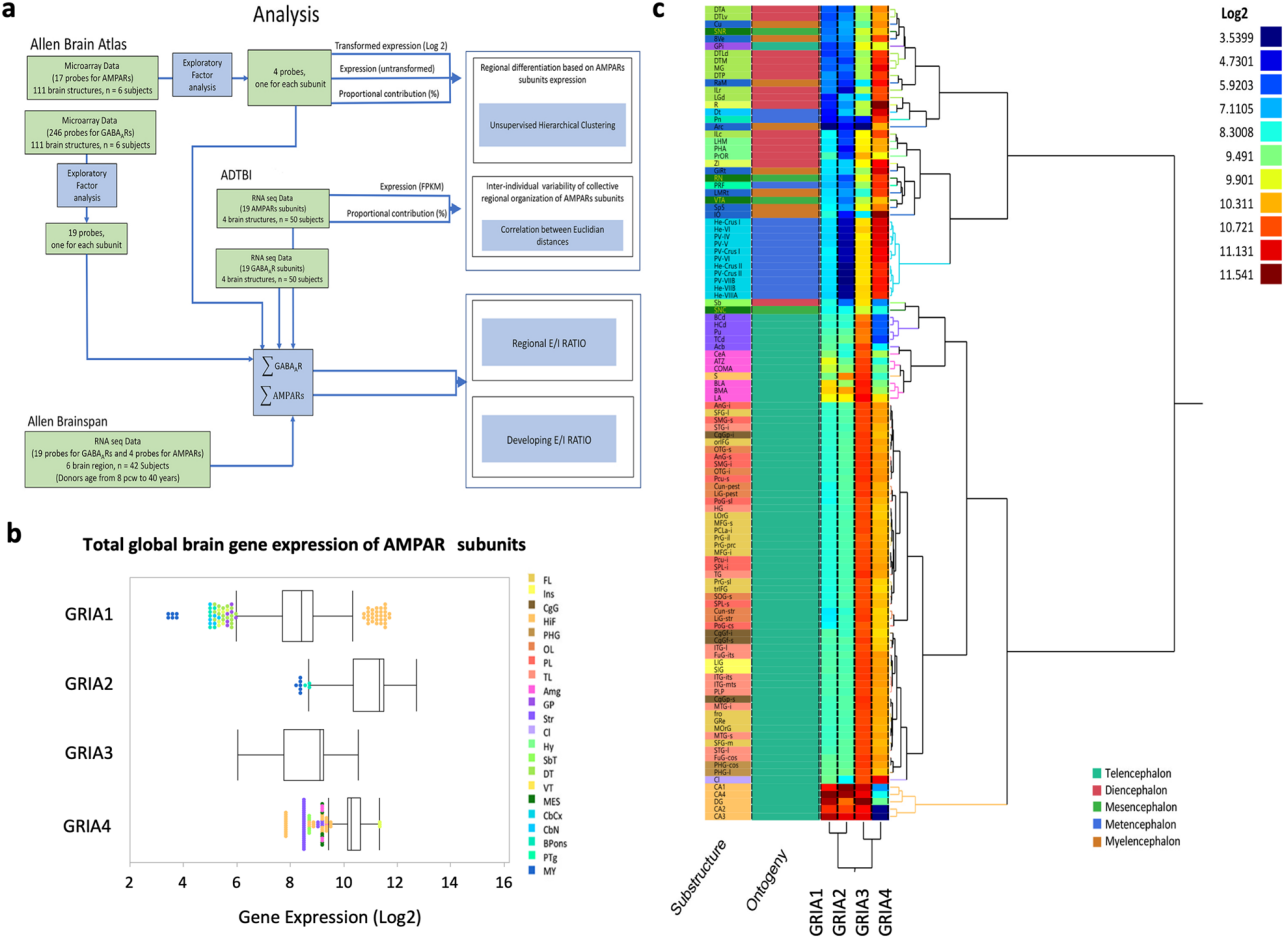
Global and regional transcriptomic analysis of AMPAR subunits (GRIA1-4) in the human brain. (**a**) Diagram detailing transcriptomic workflow. Publicly available data from the Allen Brain Atlas, Brainspan, and the ADTBI studies were filtered using exploratory factor analysis and analyzed at the global and regional levels for both GRIA1-4 expression and *t*E/I ratio patterns. (**b**) Box plots of microarray gene expression from the Allen Brain Atlas (111 substructures per 6 subjects) and corresponding percent contributions at the global level. The median is represented by the line within the box, and the first and third quartiles are represented by the ends of the box. The whiskers extend from each end of the box to the first or third quartile ± 1.5 (interquartile range). Points outside of the whiskers are outliers and color-coded according to the inset. *FL* frontal lobe, *Ins* insula, *CgG* cingulate gyrus, *HiF* hippocampal formation, *PHG* parahippocampal gyrus, *OL* occipital lobe, *PL* parietal lobe, *TL* temporal lobe, *Amg* amigdala, *GP* globus pallidus, *Str* striatum, *Cl* claustrum, *Hy* hypothalamus, *SbT* subthalamus, *DT* dorsal thalamus, *Vt* ventral thalamus, *MES* mesencephalon, *CbCx* cerebellar cortex, *CbN* cerebellar nuclei, *Bpons* basal part of the pons, *PTg* pontine tegmentum, *MY* myelencephalon. (**c**) Two-way unsupervised Ward’s hierarchical clustering of microarray data from the Allen Brain Atlas for analysis of substructures. Major brain regions were separated by Log2 gene expression, and subunits were clustered according to their regional expression levels. Structures are color coded as in b, and ontogeny is detailed by the inset. For substructure abbreviations, please see Supplementary Data 2.

Our analysis was performed at different levels using publicly available data (Fig. 1a). First, we examined major relationships between AMPAR subunit expression levels across a wide variety of structures from a microarray dataset from the Allen Brain Institute. This analysis, although low in number of subjects (n = 6), was extensive in its anatomical coverage, which ranged across 111 brain structures^30.^ To analyze inter-individual differences, we utilized RNA-sequencing (RNA-Seq) data from the Aging, Dementia, and Traumatic Brain Injury (ADTBI) study (https://aging.brain-map.org/), which included a much higher number of individuals (n = 50), but was limited to only four regions: the hippocampus (*HIP*), temporal cortex (*TCx*), parietal cortex (*PCx*), and forebrain white matter (*FWM*). Together, these complementary analyses created a signature of AMPAR subunit expression in the healthy human brain. To determine whether transcripts for AMPAR subunits and GABA_A_R subunits were balanced, we then calculated and analyzed the ratio between AMPAR and GABA_A_R subunit expression at both the whole brain and structural levels. This generated an estimate of the most basic signature of the transcriptional E/I (*t*E/I) ratio. Finally, to investigate the usefulness of these signatures, we applied our method to single cell data from phenotypically characterized interneurons in mice and to RNA-sequencing data from the Allen Brainspan study that ranged from early embryonic stages to adulthood in humans.

## Results

### Global and region-specific expression patterns of AMPAR subunits

We first analyzed the microarray data from the Allen Brain Atlas at the whole brain level. From this data, we selected the most representative probe for each gene (Supplementary Data 1) using exploratory factor analysis (Fig. 1a; Supplementary Fig. S1 for selection process) and divided the brain into major regions, structures, and substructures following the Allen Brain Atlas’ nomenclature (Supplementary Data 2). The initial global analysis after adjusting by age (Supplementary Data 3; Supplementary Fig. S2) revealed high expression levels (Log_2_ > 4) for all four subunits (Fig. 1b). GRIA2 was the most expressed AMPAR subunit in the whole brain followed by GRIA4, GRIA1 and GRIA3 (Fig. 1b). However, there was high variability across brain regions in expression levels for all four subunits, particularly for GRIA1 and GRIA4 (Supplementary Fig. S2). For example, structures such as the hippocampal formation (*HiF*; *CA1-4* and *DG*) and the internal globus pallidus (*GP*_*i*_) were outliers at the global expression level for GRIA1. A nested ANOVA analysis of gene expression, where substructures are nested within structures to account for substructure dependencies, confirmed different levels of expression for all 4 subunits across major structures and across some substructures within the same region (Supplementary Data 4). To address this regional variability, unsupervised hierarchical clustering was performed to group together structures with similar expression profiles (Fig. 1c). The strength of these clusters was examined through non-parametric bootstrap resampling to calculate both the approximately unbiased probability (AU) and bootstrap probability (BP) *p* values (Supplementary Fig. S3). The structures were first split between structures of telencephalic origin and those of diencephalic, mesencephalic, metencephalic, and myelencephalic origins (AU > 0.95, Fig. 1c). Within the latter group, further divisions based on structure ontogeny were observed. Regions of metencephalic origin exhibited very low levels of GRIA3 expression (Log_2_ < 4) and high levels of both GRIA2 and GRIA4 (Log_2_ > 9) while regions originating from the diencephalon, mesencephalon, and myelencephalon exhibited lower GRIA2 expression (Log_2_ < 9.9; Fig. 1c). There was very strong evidence (AU > 0.95) for clustering of metencephalic structures at nearly all levels. Structures from these three regions were then further split between those with GRIA1 expression levels comparable to metencephalic structures (Log_2_ ≈ 8) and those with very low GRIA1 expression levels (Log_2_ < 6). However, evidence for clustering of substructures within these structures was not as robust (AU < 0.95).

By contrast, the telencephalic cluster exhibited a high degree of homogeneity, particularly within cerebral cortical regions where high levels of GRIA2 and GRIA4 (Log_2_ > 9.5) and lower levels of GRIA1 and GRIA3 (Log_2_ < 9.5) were observed (Fig. 1c). Some slight variations were introduced with the cerebral nuclei which typically exhibited lower levels of GRIA4 (Log_2_ < 9) and a structure-dependent increase in GRIA1 expression (Log_2_ > 9). However, this cluster was not as robust as that of the cortical regions with an AU ± S.E.M of only 0.942 ± 0.005 (Supplementary Fig. S3). The hippocampal formation differed greatly from all other structures with high GRIA1-3 expression (Log_2_ > 10) but low GRIA4 expression (Fig. 1c), and the evidence for this cluster was very strong (AU ± SE = 0.999 ± 0.001).

The hippocampal formation contained the substructures with the greatest fold enrichment of GRIA1 (*CA4*), GRIA2 (*DG*) and GRIA3 (*CA1*) expression (Table 1). GRIA1 was particularly enriched in the *CA4* with an expression ~ 1,000% larger than the global brain average.

**Table 1:** Human brain substructures with greatest anatomical enrichment.

Subunit	Major region	Structure	Substructure	Expression (log2)	Fold enrichment
GRIA1	Cerebral cortex	HiF	CA4	11.54	10.12
GRIA2	Cerebral cortex	HiF	Dentate Gyrus	12.63	3.31
GRIA3	Cerebral cortex	HiF	CA1	10.46	3.75
GRIA4	Tectum	VT	Reticular nucleus	11.33	2.23

The expression (log2) represents the average per brain region. The fold enrichment is the substructure’s value divided by the global average.

*HiF* Hippocampal Formation, *VT* ventral thalamus.

### Proportional contributions of AMPAR subunits and inter-individual variability

**Figure 2:**
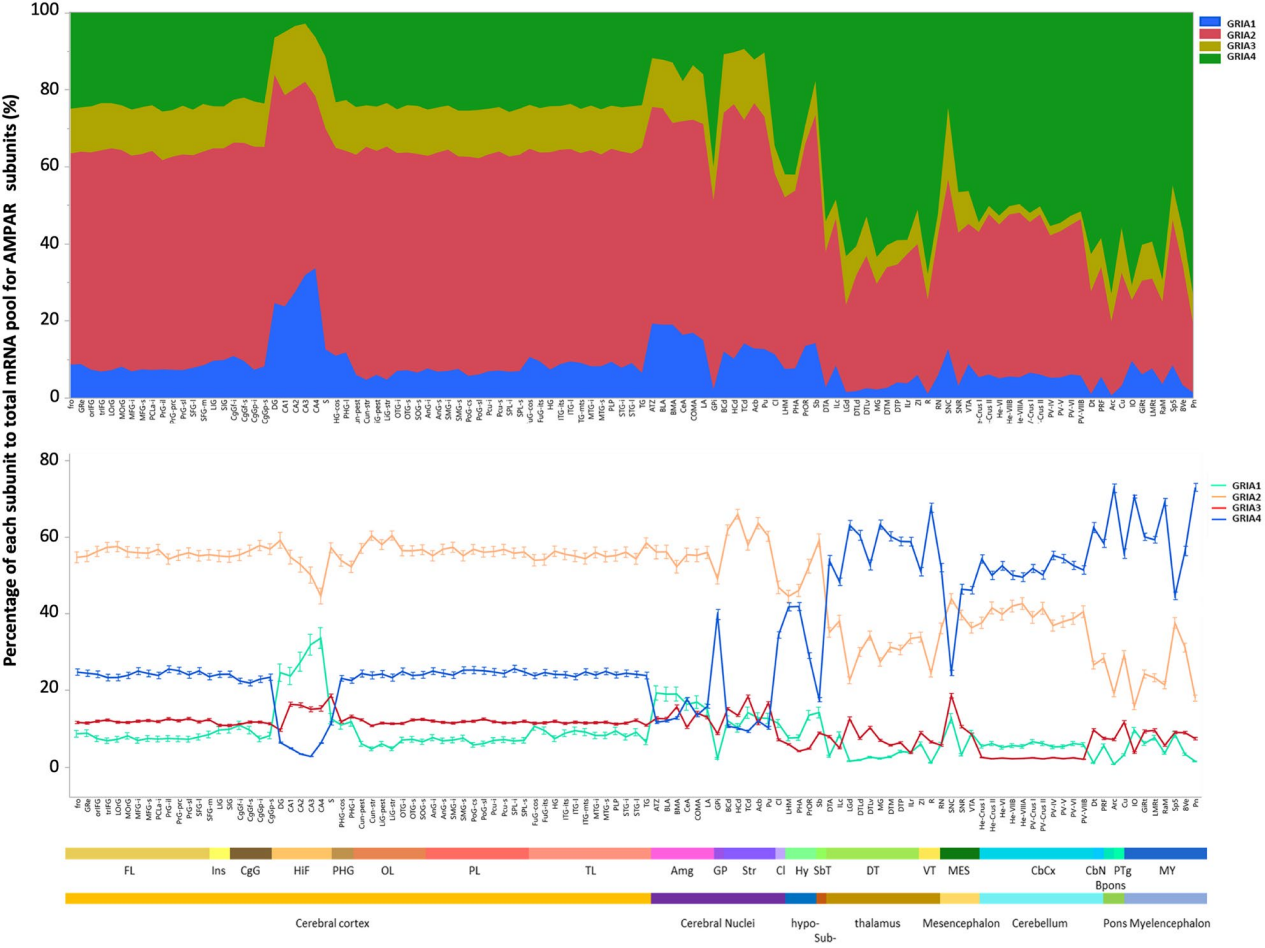
Region-specific proportional contribution of AMPAR subunits (GRIA1-4) across the brain. The area map graph (top) shows proportional contributions of each subunit as percentages to the total pool of AMPAR subunits in the brain. The line graph (bottom) of proportional contributions by each subunit more clearly shows interregional trends. (n = 6 subjects, 111 substructures) Structures are organized in a rostro-caudal order according to the Allen Brain Atlas. *FL* frontal lobe, *Ins* insula, *CgG* cingulate gyrus, *HiF* hippocampal formation, *PHG* parahippocampal gyrus, *OL* occipital lobe, *PL* parietal lobe, *TL* temporal lobe, *Amg* amigdala, *GP* globus pallidus, *Str* striatum, *Cl* claustrum, *Hy* hypothalamus, *SbT* subthalamus, *DT* dorsal thalamus, *Vt* ventral thalamus, *MES* mesencephalon, *CbCx* cerebellar cortex, *CbN* cerebellar nuclei, *Bpons* basal part of the pons, *PTg* pontine tegmentum, *MY* myelencephalon. For substructure abbreviations, please see Supplementary Data 2.

To determine the contribution of all available transcripts for AMPAR subunits per brain region, we calculated the average percentage of expression for each subunit as was previously done for GABA_A_Rs subunits^16^; that is to calculate the sum of probe intensities across AMPAR subunits and then determine to proportional contribution of the intensity of each probe to this sum. This proportional contribution is an estimate that represents the available pool of AMPAR subunits mRNA in each brain region, structure, or substructure, and normalizes distinct levels of expression between different brain areas (Fig. 2). As before, the cerebral cortex was highly homogenous with a greater contribution from GRIA2. GRIA1 contributed more in the hippocampus and cerebral nuclei, and noncortical regions were more variable and dominated by GRIA4 (Fig. 2). Further analysis revealed opposing expression patterns between some subunits. For example, the hippocampus showed a gradual decrease in the proportional contribution of GRIA2 from the *DG* to *CA4* that was paralleled by a gradual increase in the contribution of GRIA1 (Fig. 2). Similarly, GRIA4 and GRIA2 followed opposing patterns of expression in the myelencephalon and thalamus compared to the cortex (Fig. 2).

It is important to note that microarray data is highly influenced by technical factors, and intensity levels are typically compared across experimental units for the same probe, not across probes within the same experimental unit as we are doing in this study; therefore we compared the proportional contribution of GRIA subunits in the temporal cortex using microarray (*n* = 6 subjects with 12 temporal lobe substructures per subject) with that using RNA-Seq of the ADTBI study (*n* = 50 subjects), which is a better measure of mRNA expression. Proportional contributions in both datasets showed similar relationships across GRIA1, GRIA2 and GRIA3 subunits providing support for our microarray analysis of these subunits; the proportion of GRIA4 seems to be overrepresented in microarray compared to RNA-Seq (Supplementary Fig. S4), a probable consequence of choosing the probe with higher correlations across all GRIA4 probes in the whole brain, that happened to also have the highest intensity, highlighting some of the technical limitations aforementioned (Supplementary Fig. S1).

**Figure 3:**
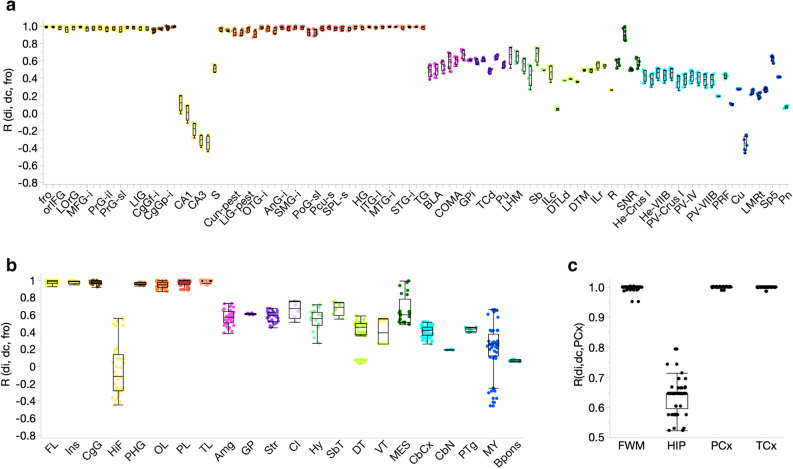
Inter-individual variability across brain regions. (**a**) Euclidian distances of AMPAR subunits per structure (111) per subject (n = 6; *d*_*i*_) were correlated against a standard (frontal operculum, *fro*; *d*_*c*_) using microarray data corrected by age. Structures are organized in a rostro-caudal order as in Fig. 2. Correlation coefficients (R) closer to one indicate higher similarity in expression patterns between the substructures and the standard. Each point is a single subject containing the collective information of all four AMPAR subunits. (**b**) Same information as in a but grouped by structure. (**c**) Euclidian distances of AMPAR subunits per structure (4) and per subject (n = 50) were correlated against the Parietal Cortex (*PCx*) using ADTBI data corrected by age. The hippocampus (*HIP*) is drastically different from the other three regions and also highly variable between subjects. *FWM*, forebrain white matter, *TCx*, Temporal cortex. Each point is a single subject.

In addition to differences in level of gene expression of isolated subunits, we also explored the variability in the collective organization of AMPAR subunits between individuals. To do so, we followed the previous approach used for GABA_A_Rs^16^. First, inter-individual variability, within and across substructures of the microarray study, was quantified by calculating the Euclidian distances (*d*) between the expression levels of GRIA subunits in each region and correlating these results. For example, the correlation coefficient (R) between the superior frontal gyrus across individuals (*di*) was higher (*di*_*SFG-SFG*_ = 0.94 ± 0.05; Mean ± SD) than in the dentate gyrus (*di*_*DG-DG*_ 0.81 ± 0.18), indicating that the dentate gyrus is more variable across individuals than cortical regions (Supplementary Fig. S5). To extend the analysis and compare brain regions at the level of substructures the frontal operculum (*fro*), which is the frontal-most structure displayed in the Allen Atlas, was chosen to construct a reference (consensus) against which all other 110 substructures were compared (*dc*_*Fro*_). Each substructure from each subject was then measured against this reference (Fig. 3a and Supplementary Fig. S6). Cerebral cortical structures were mostly homogenous with correlation coefficients close to one, indicating a high similarity to other cerebral structures and to the frontal operculum (Fig. 3a and Supplementary Data 6 for statistical analyses). Hippocampal structures, as expected, differed greatly from other cerebral regions (*r* < 0.6) and were more variable between individuals (Fig. 3a). However, the microarray study included only six subjects, so we turned to the ADTBI study (n = 50 subjects) to examine inter-individual variability more accurately. For this analysis, the parietal cortex was chosen as the reference structure against which the other three structures were compared. The parietal and temporal cortices were identical with virtually no global variability between all 50 subjects (Fig. 3c). Surprisingly, unlike what was previously observed for GABA_A_R subunits^16^, the forebrain white matter was very similar to the cortices with low inter-individual variability (Fig. 3c). The hippocampus, in agreement with the microarray data, was very different from the other structures and highly variable between subjects (Fig. 3c). This difference arose primarily out of a higher proportional contribution of GRIA1 in the hippocampus as opposed to the other three regions.

### Global and region-specific patterns of transcriptional excitation–inhibition ratio markers

**Figure 4:**
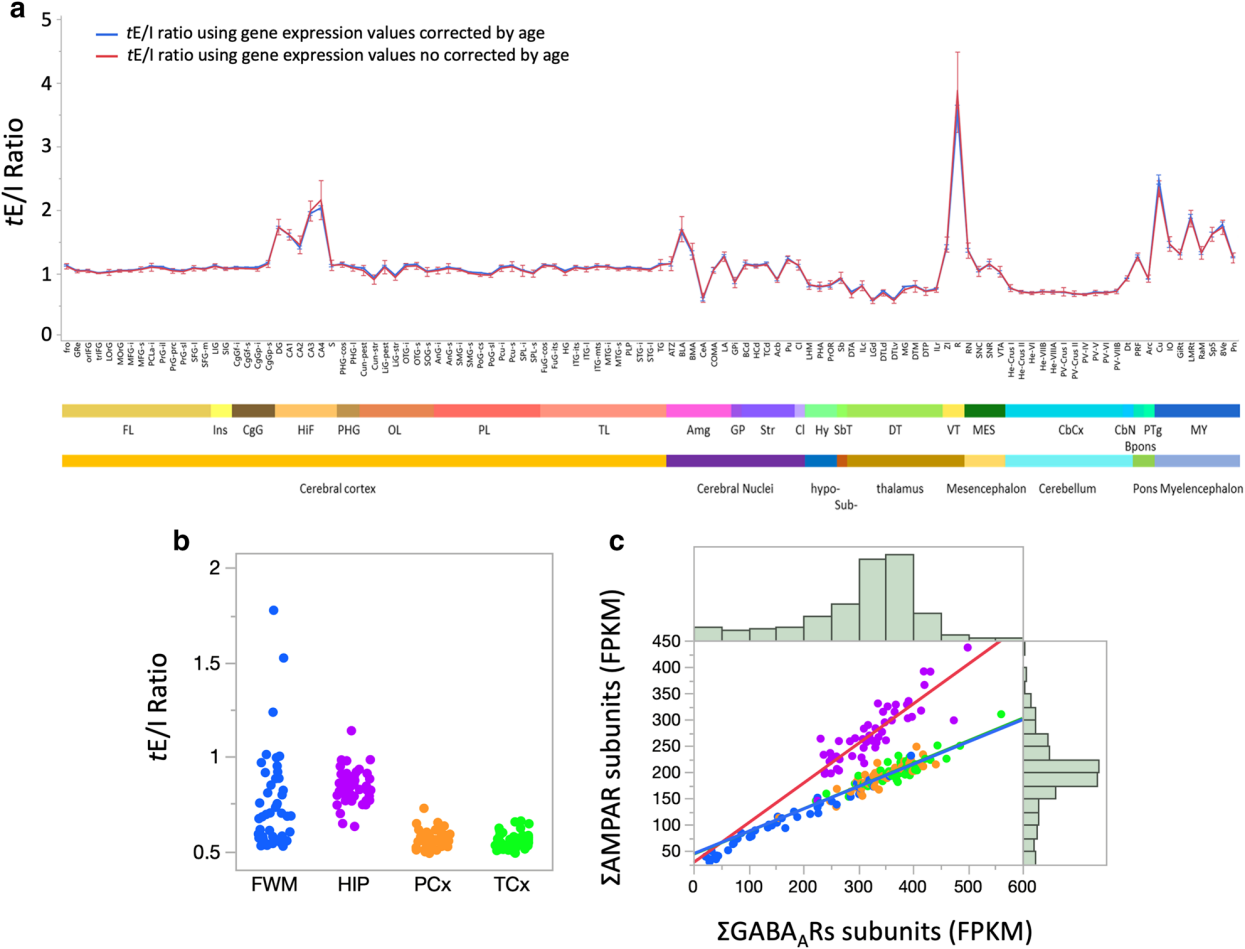
Structural differences in transcriptional E/I ratio in the human brain. (**a**) Mean ± S.E.M. of the *t*E/I (total probe intensity of AMPAR (ΣAMPARs) subunits over total probe intensity of GABA_A_R (ΣGABA_A_Rs) subunits) across the whole human brain. The ratios before (red) and after age correction (blue) were calculated for each region and were not different within brain structures. Structures were ordered along the anteroposterior axis. (**b**) The *t*E/I ratio calculated from the ADTBI RNA-Seq data. Each point represents a single subject. (**c**) Plotting total AMPAR subunit against total GABA_A_R subunit expression levels from ADTBI RNA-Seq data. Strong positive linear correlations were detected within all four structures (*p* < 0.001 for all), though with different slopes. Histograms represent the data distribution for the cohort in either axis.

**Figure 5:**
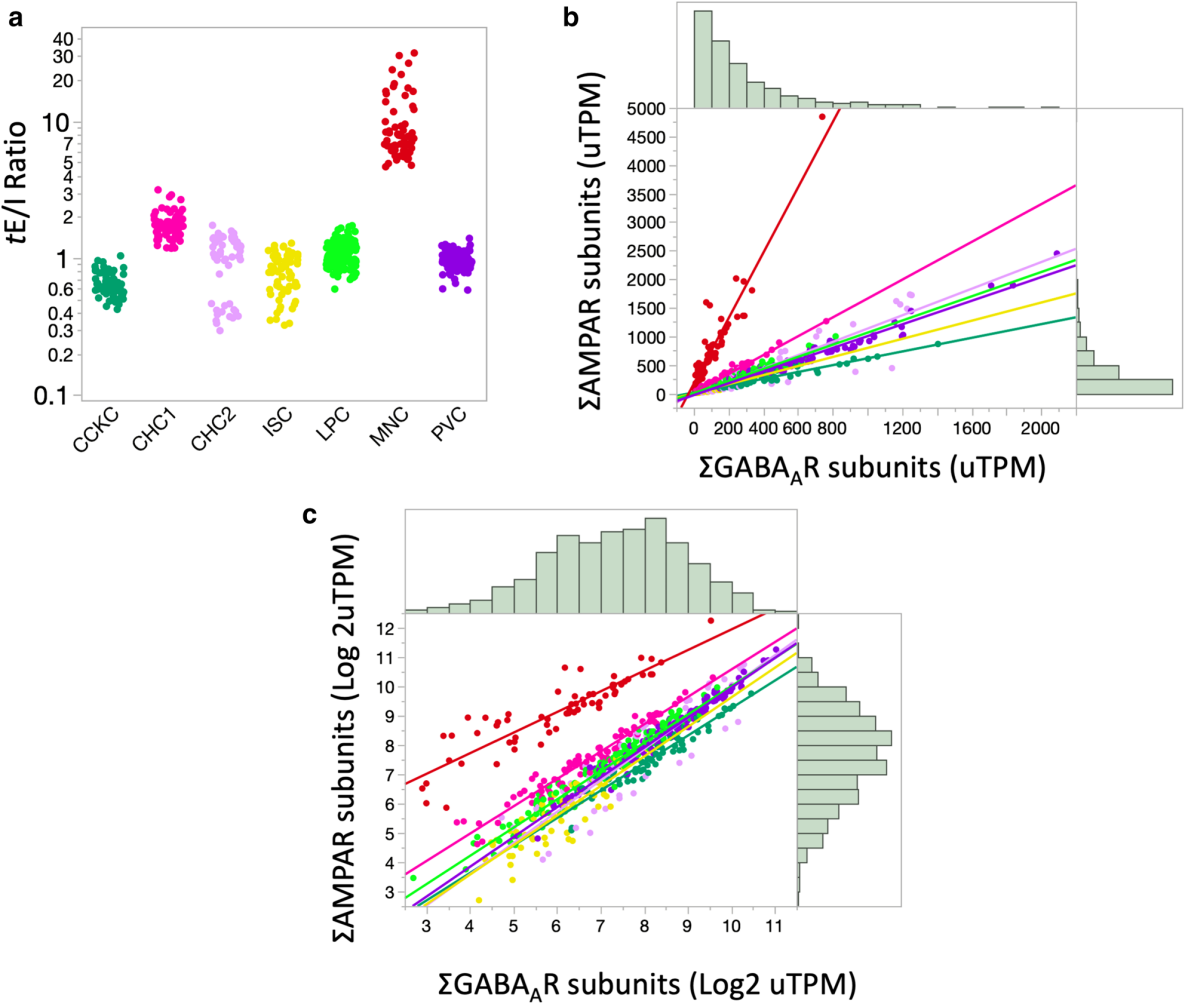
Cell-type differences in transcriptional E/I ratio in the mouse brain. (**a**) Transcriptional E/I ratio of phenotypically identified GABAergic cell-type^35^. Ratios were plotted on a logarithmic scale. Each point represents a single cell nucleus analyzed. (**b**, **c**) Plotting of total AMPARs expression against total GABA_A_Rs subunits expression using normalized data as downloaded (**b**) or after Log2 transformation to normalize the distribution (**c**). Continuous lines show significant Pearson correlations for all cell-types (*p* < 0.001). Histograms represent the data distribution for the cohort in either axis.

To combine these results with the expression patterns in GABA_A_Rs subunits we previously uncovered, we used the microarray gene expression data as a marker of the *t*E/I ratio by dividing the sum of probe intensities across AMPAR subunits by the sum of probe intensities across GABA_A_R subunits (Supplementary Data 7). This is the most basic and non-subunit specific estimation of the transcriptional foundations of the E/I balance based on gene expression for the principal gates of excitation and inhibition. Because age correction may change GABA_A_Rs and AMPARs differently and lead to distinct ratios in each subject, we compared the *t*E/I ratio using gene expression levels with and without age correction. Both ratios were linearly correlated (R^2^ = 0.789; *p* < 0.0001) and no significant difference in each substructure was observed between both ratios, although more dispersion was observed using non-corrected data (*p* > 0.29 in all cases by Welch’s testing means equal, allowing SD not equal, Fig. 4 and Supplementary Fig. S7). For simplicity, we decided to use the *t*E/I based on non-adjusted gene expression values. Expression patterns across cerebral cortical regions were relatively homogenous with *t*E/I ratios close to one (Fig. 4a). Similarly, cerebellar regions exhibited highly homogenous *t*E/I ratios though these were lower than those found in the cerebral cortex (Fig. 4a). Hippocampal structures exhibited a higher *t*E/I ratio overall compared to the rest of the brain, apart from the reticular nucleus (Supplementary Data 7). Within the hippocampus, the *t*E/I ratio gradually decreased from *DG* to *CA2* and then increased from *CA3* to *CA4* where AMPAR subunit expression was nearly double that of GABA_A_Rs subunit expression (Fig. 4a). Cerebral nuclei and thalamic regions were more variable with more caudal regions tipping towards greater inhibition (Fig. 4a). The structure with the greatest difference was the reticular nucleus in the ventral thalamus (*t*E/I_mean_ = 3.8, Fig. 4a). These observations were further supported by the ADTBI RNA-Seq dataset. The *t*E/I ratios using non-adjusted by age gene expression in the parietal and temporal cortices were remarkably homogeneous and both were significantly different from the hippocampus, which was both higher and more spread (Fig. 4b; Supplementary Data 8 for statistical analysis). No differences in the *t*E/I ratio in the temporal and parietal cortices, hippocampus or forebrain white matter by sex were observed (*p* > 0.11 double tailed *t* test; Supplementary Fig. S8). Even though the *t*E/I ratio in the forebrain white matter was more variable across individuals, the *t*E/I ratios from these structures were remarkably constant overall, indicating that AMPARs and GABA_A_Rs were highly correlated within brain structures, although with different slopes (Fig. 4c). Such strong correlations between AMPARs and GABA_A_Rs suggest that transcriptional correlations start at the cellular level. Indeed, the electrophysiological E/I (*e*E/I) balance has been demonstrated in excitatory pyramidal neurons as well as inhibitory interneurons^31–33^. However, analyzing the *t*E/I at the single cell level by single-cell RNA-Seq (sc-RNA-Seq) in humans is challenging due to the inherent low coverage of the method (approx. 10–20% of the transcriptome) and the potential mRNA degradation by postmortem interval and agonal factors^34^. Therefore, we used a high resolution sc-RNA-Seq dataset from six phenotypically identified cortical interneurons from mice^35^ to test for intra- and inter-variability of the *t*E/I ratio at the cell-type level (Fig. 5). For this analysis, we used the dataset from Paul et al.^35^ available on the gene expression omnibus (GEO; GSE92522) which provided data from anatomically and physiologically identified cortical chandelier cells (CHCs), perisomatic fast-spiking basket cells (PVBCs), long-projecting interneurons (LPCs), Martinotti cells (MNC), interneuron-selective cells (ISCs) and CCKC basket cells (CCKCs).

These cells demonstrated strong linear correlations between AMPARs and GABA_A_Rs with *t*E/I ratios averaging between 0.66 to 1.8 (Fig. 5b; Table 2). Only MNCs were significantly different with a tenfold higher *t*E/I ratio (P < 0.0001; ANOVA followed by Tukey–Kramer HSD), and CHC1 cells were significantly different from CCKC cells (P = 0.022). No other cellular types were significantly different from the others (P > 0.05).

**Table 2:** Cell-type transcriptional E/I ratios.

Cell-type	Number of cells	Mean ± SD
CCKC	64	0.66 ± 0.13
CHC1	80	1.7 ± 0.38*
CHC2	52	1.0 ± 0.43
ISC	63	0.80 ± 0.26
LPC	136	1.1 ± 0.22
MNC	62	10 ± 6.3**
PVBC	127	0.96 ± 0.13

The mean expression represents the average per cell type.

*CCKC* basket cells, *CHC1* chandelier cells 1, *CHC2* chandelier cells 2, *ISC* interneuron-selective cells, *LPC* long-projecting interneurons, *MNC* Martinotti cells, *PVBC* perisomatic fast-spiking basket cells.

*Different from CCKC (*p* = 0.022).

**Different from all other cells (*p* < 0.0001).

### *t*E/I ratio expression patterns change during development

**Figure 6:**
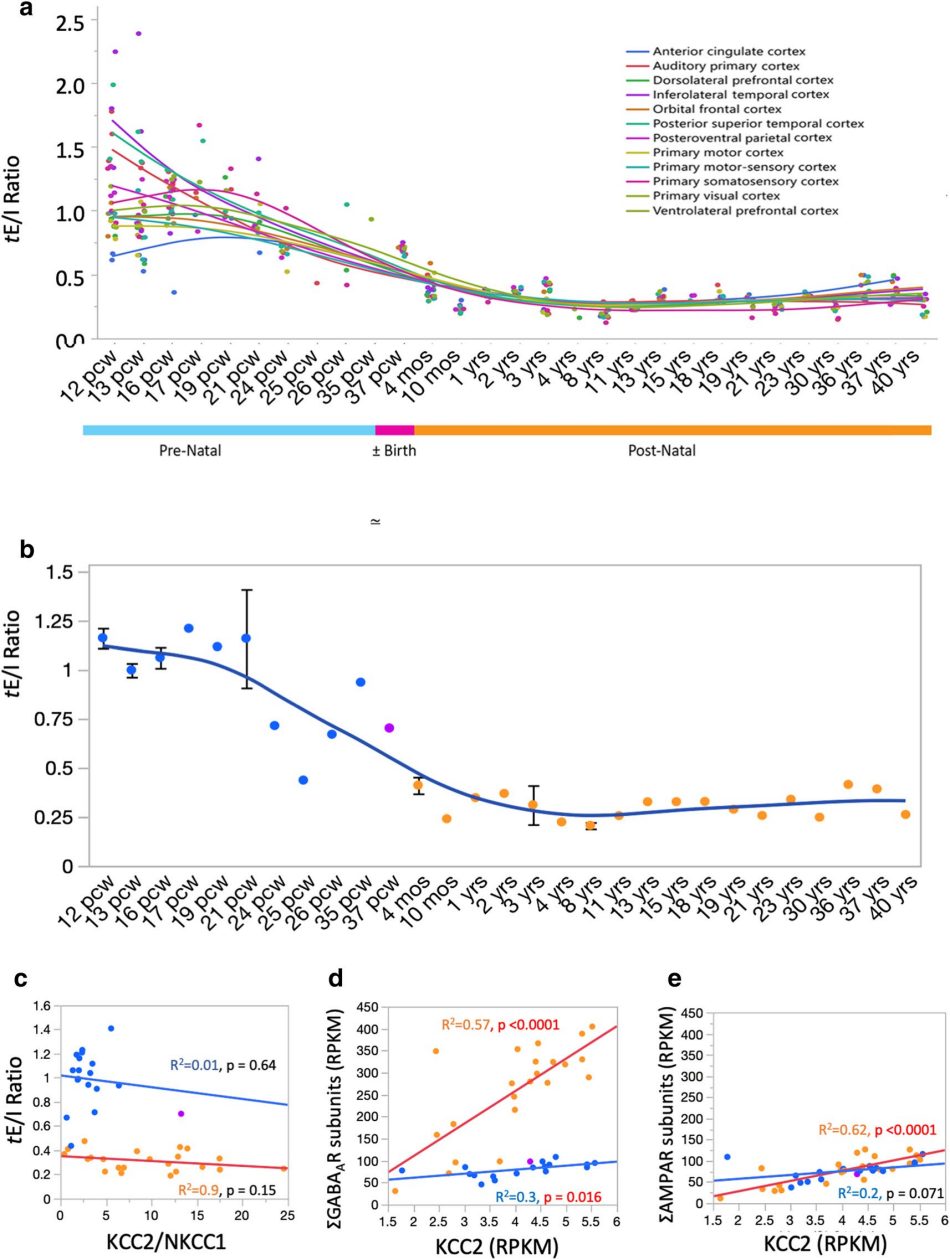
*t*E/I ratio and KCC2/NKCC1 ratio in the developing human brain. (**a**) *t*E/I ratios were calculated in different cortical regions against age (from 12 postconceptional weeks to 40 years, shown as categorical values in the plot) using data from the Allen Brainspan project (*n* = 40 subjects). Each point represents a single cortical region per subject. Spline smooth curves, here and in (**b**), show the principal trend in values for AMPA and GABA_A_ receptors. Please see Supplementary Data 9 for detailed number of subjects per region. (**b**) The graph shows the mean ± S.E.M. of cortical *t*E/I in each subject per region. The cortical *t*E/I in each subject is the average of all cortical regions available for each subject. (**c**) Lack of correlation between the cortical *t*E/I ratio in each subject and the KCC2/NKCC1ratio. KCC2 is the potassium-chloride transporter member 5 produced by the SLC12A5 gene and its expression increases during development, while NKCC1 is the Na–K–Cl cotransporter produced by SLC12A2 gene. The relationship between these transporters define the direction of chloride permeability in GABA_A_Rs. (**d**, **e**) KCC2 expression is linearly correlated with the increase of GABA_A_Rs (**d**) and AMPARs (**e**) after birth. Points are color coded as prenatal (blue), near birth (magenta), or post-natal (orange). Linear correlations are for data grouped as pre and postnatal development.

To demonstrate the flexibility and capabilities of our approach, we applied our workflow to developmental data gathered by Allen Brainspan focusing on the cortex since it has more data than the other structures studied in that dataset (Supplementary Data 9). The *t*E/I ratio across cortical regions was initially spread across a large range, but all structures reached a relatively stable *t*E/I ratio by one postnatal year (Fig. 6a). Interestingly, the *t*E/I ratio in the cortex using the Brainspan dataset was smaller than the one in the ADBTI study, although both used RNA-Seq methods (*t*E/I = 0.31 ± 0.0061 (mean ± S.E.M) in Brainspan vs 0.5842 ± 0.0045 in ADTBI; double tailed *t* test, *p* < 0.001). During pre-natal development, some structures, namely the dorsolateral and inferolateral temporal cortices, auditory primary cortex, and posteroventral parietal cortex exhibited high *t*E/I ratios that rapidly declined approaching birth (Fig. 6a). Other structures, such as the primary somatosensory and anterior cingulate cortices rose slightly during early development and peaked at around 19 post-conception weeks (pcw) before declining towards the steady state observed in adulthood (Fig. 6a). Overall, the cortical *t*E/I ratio stabilizes after birth. Closer inspection of the data revealed that the developmental reduction of *t*E/I resulted from a gradual increase in GABA_A_R subunit expression levels while AMPAR subunit levels remained stable (Supplementary Fig. 9). In addition to the *t*E/I ratio, we also analyzed the patterns of expression of the sodium–potassium–chloride cotransporter (NKCC1, *SLC12A2*) and the neuron-specific chloride–potassium symporter 5 (KCC2, *SLC12A5*) which have been demonstrated to change with development and determine whether GABA_A_Rs are inhibitory or excitatory^36^. KCC2 expression increases, while NKCC1 expression decreases during development. To do so, we compared the ratio of *SLC12A5* to *SLC12A2* with the *t*E/I ratio and found that during prenatal development, the higher *t*E/I ratio corresponded to a low *SLC12A5*/*SLC12A2* ratio while postnatal development exhibited no relationship between the two ratios (Fig. 6c). KCC2 expression is the main driver of variability in the KCC2/NKCC1 ratio. Interestingly, KCC2 gene expression was linearly correlated with the global expression of GABA_A_R and AMPAR subunits.

## Discussion

Our clustering analysis indicates that the co-expression patterns of AMPARs subunits is region specific according to the ontogenic origin of the brain structures. It is quite remarkable that with the expression of only 4 genes, major brain regions could be separated by unsupervised clustering analysis. This suggests that AMPARs may act as strong gene markers in synaptic components previously described by bioinformatics analysis^37–40^. Our microarray analysis fit the clustering scheme created by Gold et al. in the rodent model using in situ hybridization^41^, though with some differences. For instance, while the higher contribution of GRIA1 in the amygdala and hippocampus relative to other cerebral structures was mirrored in our analysis, it was to a much lesser extent, only 20–30% as compared to the 50% reported. Further, the cerebellum exhibited a high GRIA4 profile that is inconsistent with any of the reported clusters. On the other hand, cortical regions aligned fully with the pattern of ~ 50% GRIA2 contribution. While data from mice showed GRIA3 and GRIA4 enrichment relative to GRIA1 in the thalamus, our analysis found only GRIA4 enrichment. Both differences may be related to the high anatomical specificity of structures in hybridization studies compared to the microarray data that we used for our analysis, or it could be due to divergent characteristic between species^42^.

One potential drawback of our microarray analysis is the low number of samples which reduces the statistical validity of our findings. However, our main aim for this microarray data was to generate a transcriptional signature of structural clusters based on the relative expressions of the different GRIA subunits. For this purpose, we utilized bootstrapping to measure the strength of our clusters. As a result, we discovered very strong evidence for upper-level divisions between cortical regions of telencephalic origin and regions of myelencephalic, metencephalic, and diencephalic origin, as well as the existence of distinct structural clusters, such as the hippocampal formation. While these results are promising, additional samples would boost the power of this study.

Our observed region-specificity in AMPAR subunit expression correlates with the differing complexity in pathways and cytoarchitecture and with embryonic origin. Indeed, previous studies have found that a large selection of genes exhibit region-specific expression largely dependent on their embryonic structure of origin^38,43^. For brain structures with recurrent cytoarchitecture, such as the cerebral and cerebellar cortices^44^, AMPAR subunit expression patterns are highly stable well into adulthood. This shared pattern may result from a shared embryonic origin such as the pallial regions for cortical glutamatergic cells^45^ and the rhombic lip for the glutamatergic cells in the cerebellar cortex^46^. In contrast, the more heterogenous patterns observed in deep cerebral nuclei, thalamic nuclei, and mesencephalic structures are congruent with more heterogenous cytoarchitecture of these regions and may underly the diverse modulatory functions of these nuclei^47^. Recent studies have begun to demonstrate the importance of using functional rather than neuroanatomical principles for the analysis of synaptic proteins^48^; similar analytical approaches with gene expression may help to explain the heterogenous expression of AMPA receptor subunits in the thalamic nuclei and mesencephalic structures in future studies.

Interestingly, the hippocampal formation differed greatly from the cerebral cortices, exhibited the highest expression levels of all AMPAR subunits compared to all the other brain structures, and showed the largest variability across individuals when using the correlation of Euclidian distances between subunits. The heightened expression may result from the unique neurogenesis in the hippocampus^49^, the rapid regulation of synaptic AMPA receptors necessary for its role in learning^50–52^ and the high neuronal density in this area. Previous studies have shown the necessity of GRIA1 subunit in long-term potentiation and contextual learning at the *CA3-CA1* synapse^52,53^ and the rapid subunit specific turnover of AMPARs in response to alterations in neuronal activity^54,55^. Moreover, the subgranular zone of the dentate gyrus is one of the only regions in the human brain where adult neurogenesis occurs^56^. As a part of this process, neural stem cells enter hippocampal circuits after modulation by various environmental and cell-intrinsic factors^57,58^. This intense plasticity suggests that hippocampal AMPAR subunit expression varies greatly depending on the subject and environment. Our analyses of both the microarray and RNA-seq studies support this hypothesis as inter-subject variability in AMPAR subunits expression is particularly high in the hippocampal formation while AMPAR subunits expression in the cerebral cortex is highly stereotypical across individuals.

A major interest in our analysis was to determine whether the mRNA for the receptors involved in establishing the E/I ratio were balanced across brain structures in human. Because many subunits of GABA_A_Rs and AMPARs follow complementary or opposing patterns of expression, we used the most conservative approach which includes all potential receptor configurations by adding transcripts for all subunits when estimating the *t*E/I ratio. Our analysis found that the total amount of AMPARs and GABA_A_Rs is highly correlated in a region-specific manner. Therefore, the *t*E/I ratio remains highly stable across substructures within major regions such as the cerebral and cerebellar cortices but varies gradually within the hippocampus and amygdala, suggesting different balances within these regions. Our results using the ADTBI study did not find sex differences for the *t*E/I ratio, suggesting that in healthy individuals, sex differences for particular GABA_A_Rs subunits like higher cortical α1 in men^59^, may be balanced by compensatory mechanisms regulating the expression of AMPARs.

Our study unfortunately cannot distinguish between synaptic and non-synaptic receptors; therefore, our inferences are limited to estimates of total transcription levels per brain structure in the microarray and ADTBI analyses, or to the cell-type level in the single cell analysis. Nevertheless, the electrophysiological activity of synaptic and non-synaptic receptors GABA_A_Rs strongly correlates within single neurons^60^, and AMPARs show high lateral mobility between non-synaptic and synaptic regions^61^ suggesting that whole transcription levels may at least partially reflect the activity-dependent need for AMPAR and GABA_A_R subunit transcription. Recent studies have also shown that electrophysiological measures of global *e*E/I by recording cortical synaptic AMPARs and GABAARs are highly correlated^62^. Future studies integrating transcriptomic with electrophysiological measures of the E/I will help to understand deviations of this balance in non-physiological states.

Our analysis also shows that the *t*E/I ratio is established near birth. While AMPAR subunits expression is relatively stable during the embryonic stages, GABA_A_R subunit expression gradually increases until stabilizing near birth reducing the *t*E/I ratio to a stable value. This indicates that GABA_A_Rs are highly important in setting the *t*E/I ratio. In fact, development of GABA_A_Rs from immature GABA_A_α2- and GABA_A_α3-enriched to mature GABA_A_α1-enriched receptors has been observed to play a major role in synaptic plasticity in the cat visual cortex during the early post-natal phases^63^ and a fundamental role in the establishment of circuits and neuronal-firing properties within the mouse cortex^64^. On the other hand, although the amount of AMPARs remains quite stable during the development, the subunit expression and receptor assembly profile change markedly leading to a shift in receptor properties. As described by Henley and Wilkinson^65^, subunit adjustment is directly related to function; for instance, GRIA1 is developmentally restricted while expression of GRIA2, which produces Ca^2+^-impermeable receptors, increases after birth guiding the activation of silent glutamatergic synapses and the consolidation of the synaptic neural network. Our findings using the Brainspan dataset are in line with these descriptions (Supplementary Fig. S10), suggesting that the relationship between GRIA1 and GRIA2 are important in stabilization and regulation during brain development.

In conclusion, we show that AMPARs, as well as GABA_A_Rs, follow stereotypical patterns of expression in healthy controls; therefore, the *t*E/I ratio is remarkably stable across brain regions with recurrent cytoarchitecture. Additionally, this balance is established early after birth and remains stable through adulthood. Our analysis also shows that the *t*E/I ratio is determined at the cellular level, highly constant within neurons of the same type, and very similar across transcriptionally different cell types, with some exceptions. The stability of the *t*E/I ratio suggests that many redundant mechanisms control the concerted abundance of AMPAR and GABA_A_R subunit transcripts, and disruption of this balance may have severe impacts on proper neuron and brain function.

## Methods

### Microarray and RNA-sequencing databases

Three publicly available databases from the Allen Institute were used. For global brain analysis, normalized microarray transformed data (Log2) from the Allen Brain Atlas was downloaded from the webpage (https://human.brain-map.org). This database was generated with brain samples obtained from six subjects (five males and one female) between the ages of 24 and 57 years of age with no known neuropsychiatric or neuropathological history. Brain tissue was collected after obtaining informed consent from decedent’s next-of-kin. Institutional Review Board (IEB) review and approval was obtained for collection of tissue and non-identifying case information at the tissue banks and repositories that provided the tissue for the project. Case qualification and donor profiles can be seen in the Allen website: https://help.brain-map.org/display/humanbrain/Documentation. Only brain regions where data for all AMPAR and GABA_A_R subunits were measured were used for the analysis (111 brain structures). For gene expression measurements the Allen Institute used a custom design (by Beckman Coulter Genomics) Agilent 8 × 60 K array that includes the 4 × 44 K Agilent Whole Human Genome probe set supplemented with an additional 16,000 probes. The upper limit for detection was 2 and was used as part of the initial quality control as described in the white papers for microarray survey found at https://help.brain-map.org/display/humanbrain/Documentation. Normalization methods are also documented extensively in the white papers; briefly the data was normalized within each dissection batch through multivariate local regression fitting, each brain via quantile–quantile mapping of averages, and across all brains by aligning control samples.

For analysis of variability across individuals, normalized RNA-Seq Fragments Per Kilobase Million (FPKM) data from the Aging, Dementia, and Traumatic Brain Injury study (https://aging.brain-map.org/download/index) covered four regions from 56 healthy control subjects (35 males and 21 females) between the ages of 78 and 99 years of age. Detailed documentation of the ADTBI study, including tissue collection, tissue processing, and RNA-Sequencing and quantification, can be found at: https://help.brain-map.org/display/aging/Documentation. Briefly, RNA-Sequencing was done through Illumina TruSeq (random hexamer first strand cDNA synthesis with rRNA depletion and fragmentation) on Illumina HighSeq 2,500 using v4 chemistry, producing a minimum of 30 M 50 bp paired-end clusters per sample. FPKM gene quantifications were normalized via TbT normalization and corrected for RNA quality and batch effects.

Normalized developmental RNA-seq Reads Per Kilobase Million (RPKM) data from the BrainSpan atlas (Gencode v10 summarized to genes; https://www.brainspan.org/static/download.html) covered up to 26 regions from 42 subjects ranging from eight weeks post conception to 40 years of age. Detailed documentation of the developmental data, including Institutional Review Board approval for the tissue collection, donor and sample metadata, and transcriptome profiling methods, can be found at: https://help.brain-map.org/display/devhumanbrain/Documentation. Briefly, RNA-Sequencing was done with poly(A) selection and normalized by the addition of spike-ins.

For single cell analysis we used the processed high resolution sc-RNA-seq unique Transcripts Per Million (uTPM) dataset downloaded from GEO expression omnibus accession number: GSE92522^35^. This dataset comprises single cell transcriptomes of anatomically and physiologically characterized GABAergic neurons. Paul et al. developed combinatorial recombinase driver lines to identify and isolate 6 types of neurons. Detailed methods, including the approval for the study, can be found in the author’s manuscript^35^. Sequencing was performed with poly(A) linear amplification followed by Illumina TruSeq and normalized to a single cell’s total unique counts across all genes; approximately 9 k genes per cell were detected^35^. Our study focused on the analysis of the databases described above. All the methods to generate the original data were performed in accordance with the relevant guidelines and regulations of the respective authors’ institutions as described in the documentation accessed by the provided links.

### Analysis of AMPAR subunits

All analysis was done as previously stated for GABA_A_Rs^16^. The normalized transformed data (Log_2_) microarray data from the Allen Brain Atlas used 17 probes to measure the expression of 4 AMPARs genes, 4 probes for each GRIA1-3 and 5 probes for GRIA 4. To avoid redundant clustering due to collinearity between probes for the same gene, only the most representative probe for each gene as determined via principal axis Exploratory Factor Analysis with no rotation in JMP 14Pro were used. While the effects of sex and ethnicity were negligible on any of the datasets analyzed, corrections were made for age effects via linear regression in JMP 14. As before, all expression patterns were represented by the Mean (M) ± Standard Deviation (SD) unless otherwise stated. The proportional contributions of each subunit were expressed as percentages of untransformed (non-Log2) probe intensity level of each subunit to the sum of probe intensities across all subunits. Unsupervised hierarchical clustering was done using Ward’s minimum variance method in JMP 14. Bootstrap probability (BP) and approximately unbiased (AU) p-values were calculated through 10,000 bootstraps with *pvclust* in RStudio using ward.D2 and Euclidean distances as the clustering method and distance measures, respectively^66^. Pearson product-moment correlations were used to measure both inter-structure and inter-subject variance. Euclidean distances of each structure or subject (*d*_*i*_) were correlated against a chosen consensus (*d*_*c*_). For subjects, *d*_*c*_ was calculated from the average of all subjects; for structures, the frontal operculum (*fro*) and parietal cortex (*PCx*) were used. The Euclidean distances were calculated in RStudio as follows:

1 $$d_{i} \left( {GRIAx - GRIAy} \right) = \sqrt { (GRIAx - GRIAy)^{2} }$$

2 $$d_{i} \left( {{\mathbf{GRIAx}} - {\mathbf{GRIAy}}} \right) = \sqrt { \mathop \sum \limits_{i = 1}^{n} (GRIAx_{i} - GRIAy_{i} )^{2} }$$

where *d*_*i*_ is the Euclidean distance between two subunits, given by *x* and *y*, per subject or structure, represented by the integer *i*, and *d*_*c*_ is the consensus Euclidean distance, for all *n* subjects or structures.

### Analysis of the excitation–inhibition ratio

The *t*E/I ratio was calculated by dividing the sum of all AMPAR subunit expression by the sum of all GABA_A_R subunit expression. Pearson product-moment correlations were then run on these ratios to measure inter-structure variance. Data from the microarray (Log2, uncorrected and corrected for age), ADTBI (normalized FPKM, uncorrected and corrected for age), single-cell (uTPM), and BrainSpan (normalized RPKM) studies were all analyzed separately. In addition to the tE/I ratio, the expression of NKCC1 (*SLC12A2*), NKCC2, (*SLC12A1*) and KCC2 (*SLC12A5*) were also analyzed in a fashion similar to the AMPARs and GABA_A_Rs.

### Statistics and reproducibility

All data analysis was performed, and plots constructed, in both JMP 14 and RStudio running R3.6.3 and the mosaic package for R Markdown. All R code is available upon request.

## Supplementary information

Supplementary file1

Supplementary file2

Supplementary file3

Supplementary file4

Supplementary file5

Supplementary file6

Supplementary file7

Supplementary file8

Supplementary file9

Supplementary file10

## Data Availability

All original data was downloaded from publicly available databases with links provided in the manuscript. All data after adjustments are included in supplementary data.

## References

[1] Van Vreeswijk C, Sompolinsky H (1996). Chaos in neuronal networks with balanced excitatory and inhibitory activity. Science.

[2] Van Vreeswijk C, Sompolinsky H (1998). Chaotic balanced state in a model of cortical circuits. Neural Comput..

[3] Froemke RC, Merzenich MM, Schreiner CE (2007). A synaptic memory trace for cortical receptive field plasticity. Nature.

[4] Dorrn AL, Yuan K, Barker AJ, Schreiner CE, Froemke RC (2010). Developmental sensory experience balances cortical excitation and inhibition. Nature.

[5] Sun YJ (2010). Fine-tuning of pre-balanced excitation and inhibition during auditory cortical development. Nature.

[6] Li YT, Ma WP, Pan CJ, Zhang LI, Tao HW (2012). Broadening of cortical inhibition mediates developmental sharpening of orientation selectivity. J. Neurosci..

[7] Rubin R, Abbott LF, Sompolinsky H (2017). Balanced excitation and inhibition are required for high-capacity, noise-robust neuronal selectivity. Proc. Natl. Acad. Sci. USA.

[8] Sillito AM (1975). The contribution of inhibitory mechanisms to the receptive field properties of neurones in the striate cortex of the cat. J. Physiol..

[9] Dichter MA, Ayala GF (1987). Cellular mechanisms of epilepsy: a status report. Science.

[10] Gao R, Penzes P (2015). Common mechanisms of excitatory and inhibitory imbalance in schizophrenia and autism spectrum disorders. Curr. Mol. Med..

[11] Nelson SB, Valakh V (2015). Excitatory/inhibitory balance and circuit homeostasis in autism spectrum disorders. Neuron.

[12] Foss-Feig JH (2017). Searching for cross-diagnostic convergence: neural mechanisms governing excitation and inhibition balance in schizophrenia and autism spectrum disorders. Biol. Psychiat..

[13] Busche MA, Konnerth A (2016). Impairments of neural circuit function in Alzheimer’s disease. Philos. Trans. R. Soc. B.

[14] Ren SQ (2018). Amyloid β causes excitation/inhibition imbalance through dopamine receptor 1-dependent disruption of fast-spiking GABAergic input in anterior cingulate cortex. Sci. Rep..

[15] Tatti R, Haley MS, Swanson OK, Tselha T, Maffei A (2017). Neurophysiology and regulation of the balance between excitation and inhibition in neocortical circuits. Biol. Psychiat..

[16] Sequeira A, Shen K, Gottlieb A, Limon A (2019). Human brain transcriptome analysis finds region- and subject-specific expression signatures of GABAAR subunits. Commun. Biol..

[17] Malenka RC, Bear MF (2004). LTP and LTD: an embarrassment of riches. Neuron.

[18] Olsen RW, Sieghart W (2009). GABAA receptors: subtypes provide diversity of function and pharmacology. Neuropharmacology.

[19] Tichelaar W, Safferling M, Keinänen K, Stark H, Madden DR (2004). The three-dimensional structure of an ionotropic glutamate receptor reveals a dimer-of-dimers assembly. J. Mol. Biol..

[20] Zhao Y, Chen S, Swensen AC, Qian W-J, Gouaux E (2019). Architecture and subunit arrangement of native AMPA receptors elucidated by cryo-EM. Science.

[21] Hollmann M, Heinemann S (1994). Cloned glutamate receptors. Annu. Rev. Neurosci..

[22] Tóth K, McBain CJ (1998). Afferent-specific innervation of two distinct AMPA receptor subtypes on single hippocampal interneurons. Nat. Neurosci..

[23] Rubio ME, Wenthold RJ (1997). Glutamate receptors are selectively targeted to postsynaptic sites in neurons. Neuron.

[24] Liu SQJ, Cull-Candy SG (2000). Synaptic activity at calcium-permeable AMPA receptors induces a switch in receptor subtype. Nature.

[25] Liu SJ, Cull-Candy SG (2002). Activity-dependent change in AMPA receptor properties in cerebellar stellate cells. J. Neurosci..

[26] Salpietro V (2019). AMPA receptor GluA2 subunit defects are a cause of neurodevelopmental disorders. Nature Communications.

[27] Thorns V, Mallory M, Hansen L, Masliah E (1997). Alterations in glutamate receptor 2/3 subunits and amyloid precursor protein expression during the course of Alzheimer’s disease and Lewy body variant. Acta Neuropathol..

[28] Rubio MD, Drummond JB, Meador-Woodruff JH (2012). Glutamate receptor abnormalities in schizophrenia: implications for innovative treatments. Biomol. Ther..

[29] Eastwood SL (1995). Decreased expression of mRNAs encoding non-NMDA glutamate receptors GluRl and GluR2 in medial temporal lobe neurons in schizophrenia. Mol. Brain Res..

[30] Hawrylycz MJ (2012). An anatomically comprehensive atlas of the adult human brain transcriptome. Nature.

[31] Xue M, Atallah BV, Scanziani M (2014). Equalizing excitation–inhibition ratios across visual cortical neurons. Nature.

[32] He H, Shen W, Zheng L, Guo X, Cline HT (2018). Excitatory synaptic dysfunction cell-autonomously decreases inhibitory inputs and disrupts structural and functional plasticity. Nat. Commun..

[33] Campanac E (2013). Enhanced intrinsic excitability in basket cells maintains excitatory-inhibitory balance in hippocampal circuits. Neuron.

[34] Crow M, Gillis J (2018). Co-expression in single-cell analysis: saving grace or original sin?. Trends Genet..

[35] Paul A (2017). Transcriptional architecture of synaptic communication delineates GABAergic neuron identity. Cell.

[36] Ben-Ari Y, Gaiarsa J-L, Tyzio R, Khazipov R (2007). GABA: a pioneer transmitter that excites immature neurons and generates primitive oscillations. Physiol. Rev..

[37] Kang HJ (2011). Spatio-temporal transcriptome of the human brain. Nature.

[38] Kirsch L, Chechik G (2016). On expression patterns and developmental origin of human brain regions. PLoS Comput. Biol..

[39] Colantuoni C (2011). Temporal dynamics and genetic control of transcription in the human prefrontal cortex. Nature.

[40] Pletikos M (2014). Temporal specification and bilaterality of human neocortical topographic gene expression. Neuron.

[41] Gold SJ, Ambros-Ingerson J, Horowitz JR, Lynch G, Gall CM (1997). Stoichiometries of AMPA receptor subunit mRNAs in rat brain fall into discrete categories. J. Comp. Neurol..

[42] Hodge RD (2019). Conserved cell types with divergent features in human versus mouse cortex. Nature.

[43] Zapala MA (2005). Adult mouse brain gene expression patterns bear an embryologic imprint. PNAS.

[44] Nieuwenhuys R, Voogd J, van Huijzen C (2008). The Human Central Nervous System: A Synopsis and Atlas.

[45] Montiel JF, Aboitiz F (2015). Pallial patterning and the origin of the isocortex. Front. Neurosci..

[46] Leto K (2016). Consensus paper: cerebellar development. Cerebellum.

[47] Sherman SM (2016). Thalamus plays a central role in ongoing cortical functioning. Nat. Neurosci..

[48] Roy M (2018). Proteomic analysis of postsynaptic proteins in regions of the human neocortex. Nat. Neurosci..

[49] Lee E, Hygon S (2009). Adult hippocampal neurogenesis and related neurotrophic factors. BMB Rep..

[50] Malenka RC (2003). Synaptic plasticity and AMPA receptor trafficking. Ann. N. Y. Acad. Sci..

[51] Lee H-K, Kirkwood A (2011). AMPA receptor regulation during synaptic plasticity in hippocampus and neocortex. Semin. Cell Dev. Biol..

[52] Mitsushima D, Ishihara K, Sano A, Kessels HW, Takahashi T (2011). Contextual learning requires synaptic AMPA receptor delivery in the hippocampus. PNAS.

[53] Terashima A, Suh YH, Isaac JTR (2019). The AMPA receptor subunit GluA1 is required for CA1 hippocampal long-term potentiation but is not essential for synaptic transmission. Neurochem. Res..

[54] Hou Q, Gilbert J, Man H-Y (2011). Homeostatic regulation of AMPA receptor trafficking and degradation by light-controlled single synaptic activation. Neuron.

[55] De Paola V, Arber S, Caroni P (2003). AMPA receptors regulate dynamic equilibrium of presynaptic terminals in mature hippocampal networks. Nat. Neurosci..

[56] Ming G, Song H (2011). Adult neurogenesis in the mammalian brain: significant answers and significant questions. Neuron.

[57] Kempermann G, Song H, Gage FH (2015). Neurogenesis in the adult hippocampus. Cold Spring Harb. Perspect. Biol..

[58] Toda T, Parylak SL, Linker SB, Gage FH (2019). The role of adult hippocampal neurogenesis in brain health and disease. Mol. Psychiatry.

[59] Pandya M (2019). Sex- and age-related changes in GABA signaling components in the human cortex. Biol. Sex Diff..

[60] Glykys J, Mody I (2007). The main source of ambient GABA responsible for tonic inhibition in the mouse hippocampus. J. Physiol..

[61] Chater TE, Goda Y (2014). The role of AMPA receptors in postsynaptic mechanisms of synaptic plasticity. Front. Cell. Neurosci..

[62] Scaduto P, Sequeira A, Vawter MP, Bunney W, Limon A (2020). Preservation of global synaptic excitatory to inhibitory ratio during long postmortem intervals. Sci. Rep..

[63] Chen L, Yang C, Mower GD (2001). Developmental changes in the expression of GABAA receptor subunits (α1, α2, α3) in the cat visual cortex and the effects of dark rearing. Mol. Brain Res..

[64] Fagiolini M (2004). Specific GABAA circuits for visual cortical plasticity. Science.

[65] Henley JM, Wilkinson KA (2016). Synaptic AMPA receptor composition in development, plasticity and disease. Nat. Rev. Neurosci..

[66] Suzuki R, Shimodaira H (2006). Pvclust: an R package for assessing the uncertainty in hierarchical clustering. Bioinformatics.

