# Supplementary material for: Regional transcriptome analysis of AMPA and GABAA receptor subunit expression generates E/I signatures of the human brain
Source: Sci Rep. 2020 Jul 9;10:11352. doi: 10.1038/s41598-020-68165-1 (PMC7347860; doi:10.1038/s41598-020-68165-1)
Supplement: Supplementary file 1 — Supplementary file1 [file 41598_2020_68165_MOESM1_ESM.pdf]

Regional transcriptome analysis of AMPA and $GABA_A$ receptor subunit expression generates E/I signatures of the human brain

The transcriptional E/I ratio in the healthy and developing human brain

Kevin Shen, Tommaso Zeppillo, and Agenor Limon

Supplementary Material

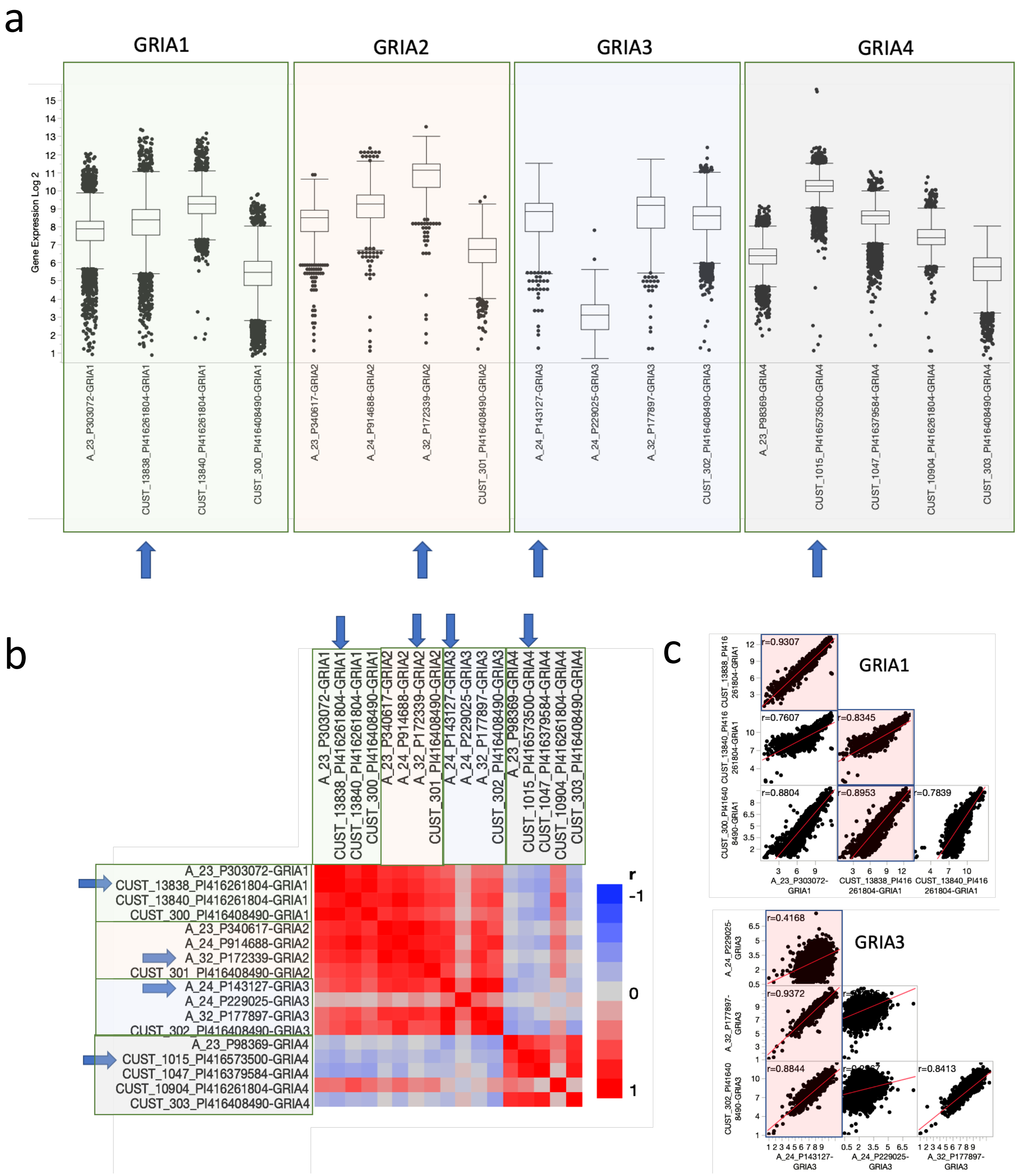

Supplementary Fig. 1. Probes for AMPARs subunits in microarray data. a, Global gene expression for each of the probes available in the Allen Atlas human brain. Each point is an outlier value of a single substructure for each subject. The median is represented by the line within the box, and the 1st and 3rd quartiles are represented by the ends of the box. The whiskers extend from each end of the box to the 1st or 3rd quartile ± 1.5 (interquartile range) Box plots in all figures contain this information. **b,** Multivariate correlations of all the probes. Notice the high correlation between most of the probes for each subunit. **c,** Scatter plots of selected correlations used for the colormap shown in b. Probes with lower values showed lower values of correlation with probes for the same subunit. The red line indicates the linear correlation and the r value is shown as insert within each plot. Blue arrows in a and b, and shaded box indicates the most representative probes identified by exploratory factory analysis. For values of the analysis please see Supplementary Data 1.

a

Downloaded normalized Allen Microarray data

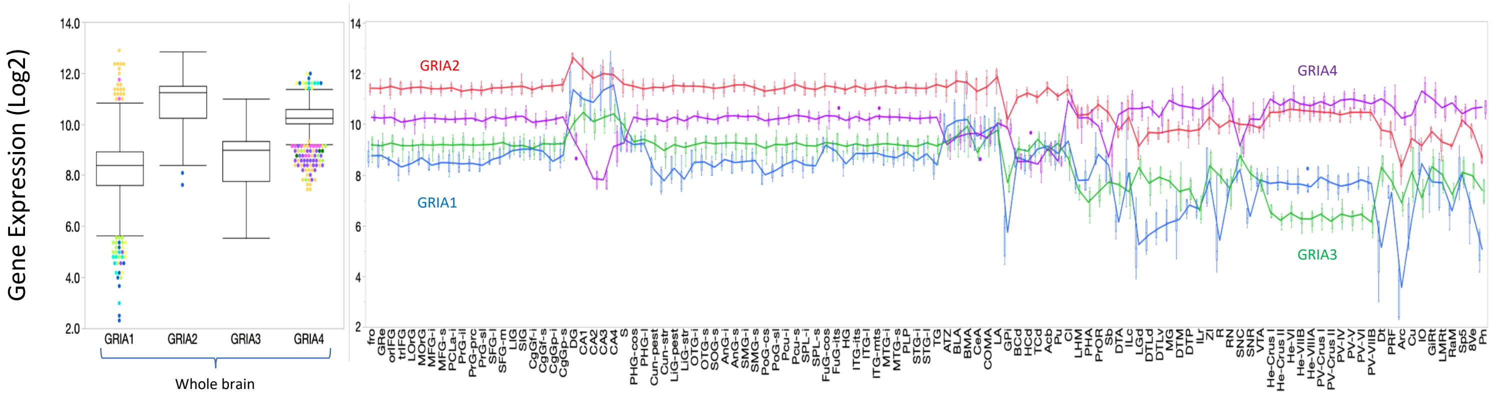

b

Downloaded normalized Allen Microarray data corrected by age

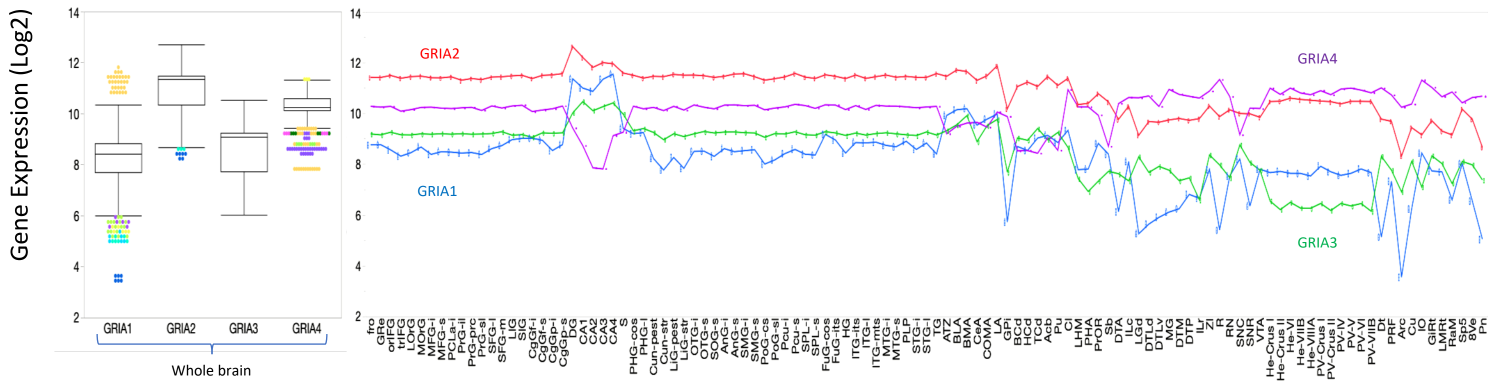

Supplementary Fig. 2. Gene expression of probes selected by exploratory factory analysis. Gene expression as downloaded from the Allen atlas (a) and corrected by age (b), of each AMPARs subunit in the whole brain (left panel) and across brain substructures (right panel). Box plots are used in all graphs. Each point represent an outlier value for each substructure in each subject. The median is represented by the line within the box, and the 1st and 3rd quartiles are represented by the ends of the box. The whiskers extend from each end of the box to the 1st or 3rd quartile ± 1.5 (interquartile range).

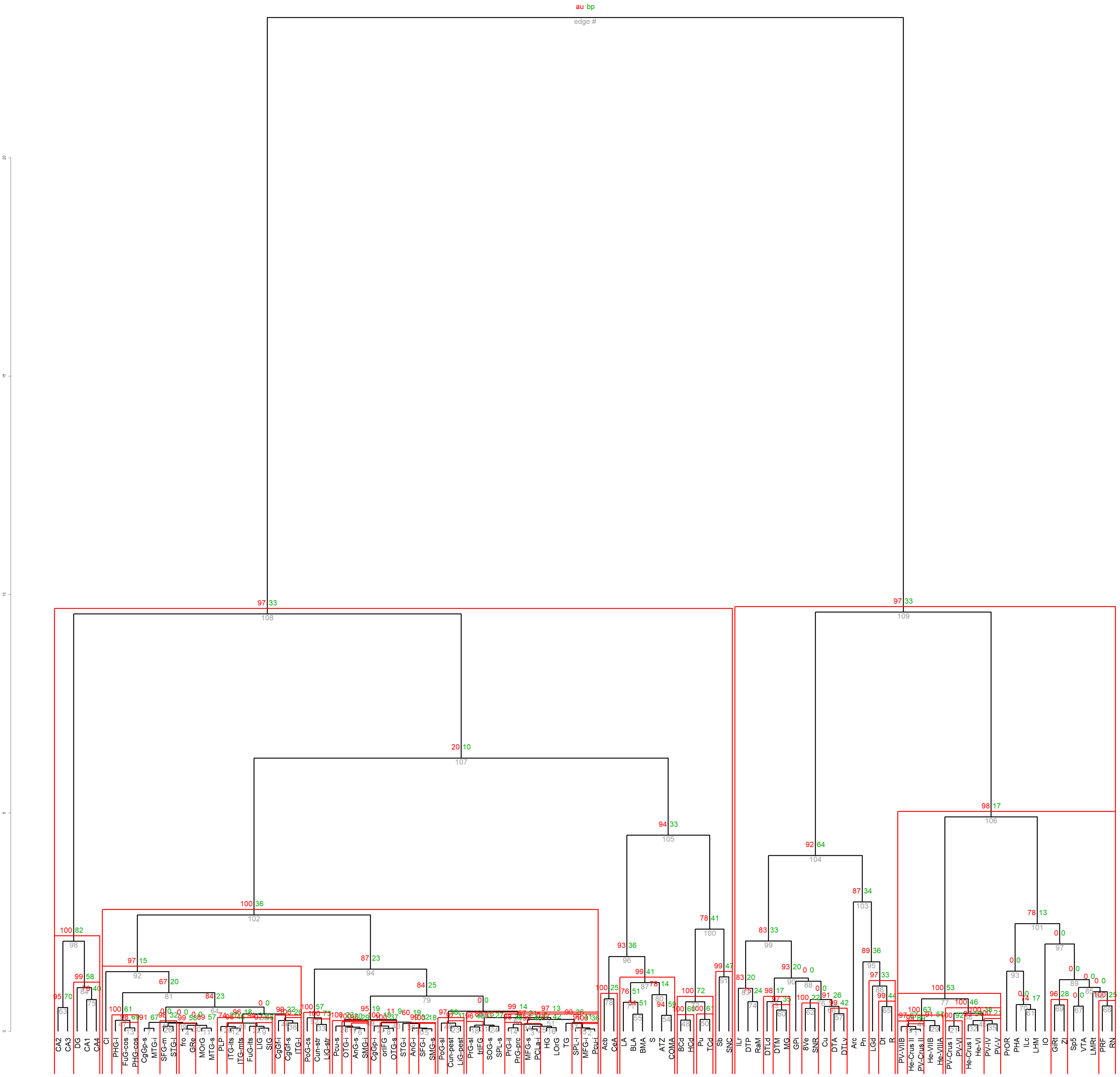

Supplementary Fig. 3. Unsupervised hierarchical clustering of structures with bootstrapping. Bootstrap probability (BP) values are in green. Approximated unbiased (AU) values are in red. Edge numbers are depicted in gray. For exact BP and AU values, see Supplementary Data 4. Clusters with strong evidence (AU > 0.95) are depicted with red rectangles.

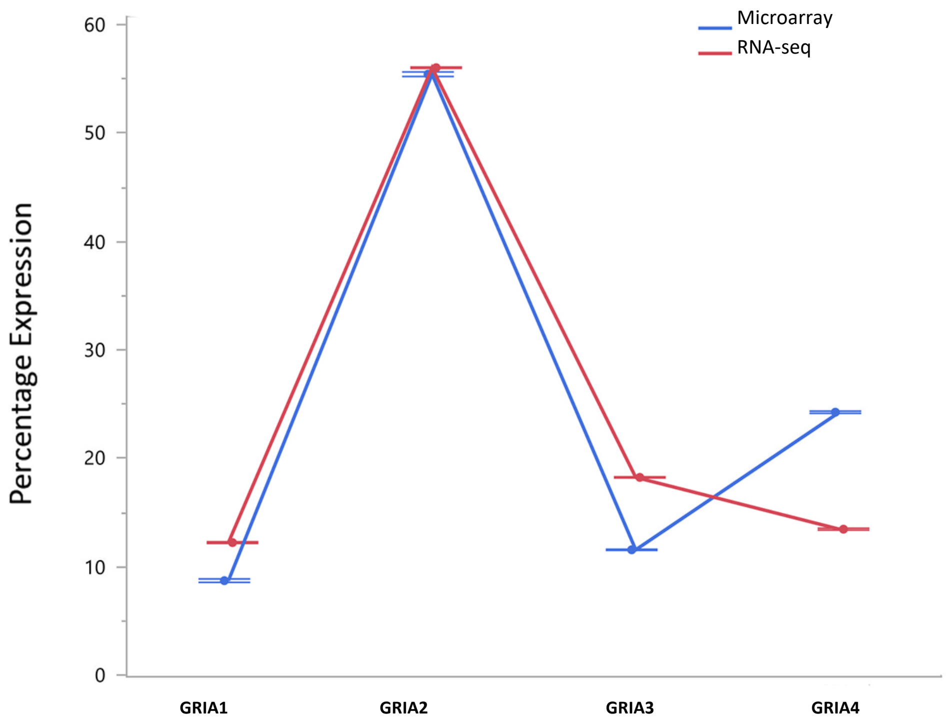

Supplementary Fig. 4. Proportional contribution to the total pool of AMPAR subunits across different datasets involving the temporal cortex. Percentage of gene expression levels of all AMPAR subunits in the temporal cortex (Mean ± SEM) of publicly available data from the Allen Brain Atlas microarray study (blue) and the Aging, and Dementia and Traumatic Brain Injury (ADTBI) study (red). To determine the contribution of all available transcripts for AMPAR subunits per brain region in the microarray study, we calculated the sum of probe intensities across AMPAR subunits and then determine to proportional contribution of the intensity of each GRIA probe to this sum. For the microarray analysis 72 independent measurements from 12 substructures and 6 control subjects were used. For the ADTBI RNA-Seq analysis the data the proportion was calculated directed from the sum of FPKM measurements for all GRIA subunits. The data was obtained from 50 subjects with non-dementia diagnosis and for whom measurements of the temporal cortex were available.

Intra- and inter-regional correlations between Euclidian distances of all AMPAR subunits across subjects

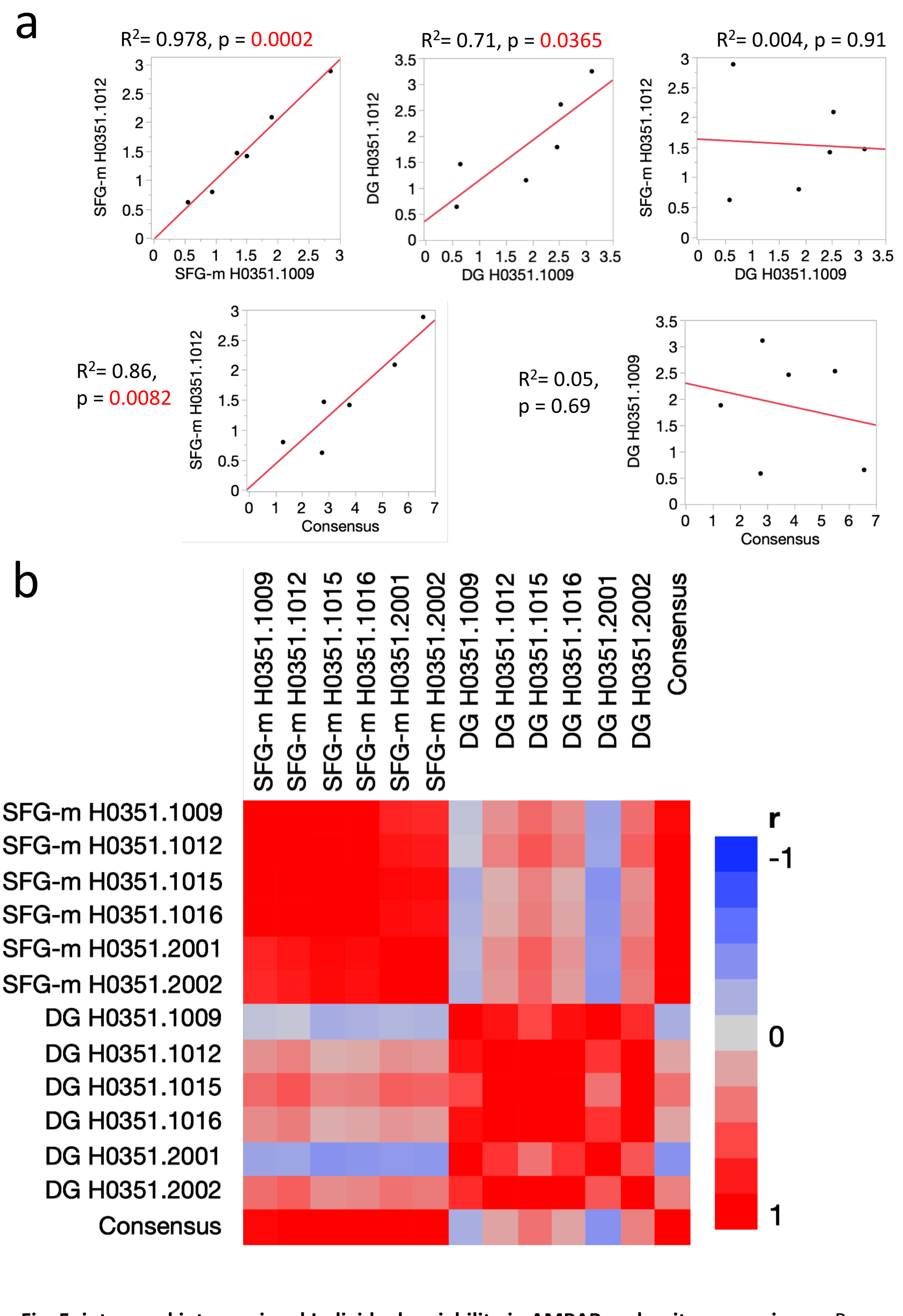

Supplementary Fig. 5. intra- and inter-regional Individual variability in AMPARs subunits expression. a, Pearson correlations between Euclidian distances across the medial superior frontal gyrus medial (SFG-m) and dentate gyrus (DG) of selected individuals. The consensus is a reference standard based on the frontal operculum, which is a cortical region and thus almost identical to the SFG-m. **b,** Colormap representing examples of Pearson correlations across subject's sets of AMPARs subunits Euclidian distances for SFG-m, DG and the consensus. The correlation across individuals within the SFG-m was 0.94 + 0.05 (Mean + SD), and across individuals within the DG was 0.81 + 0.18, indicating that the DG is more variable across individuals. The correlations between each subject's SFG-m and DG with the consensus was 0.97 + 0.08 and 0.09 + 0.32, respectively.

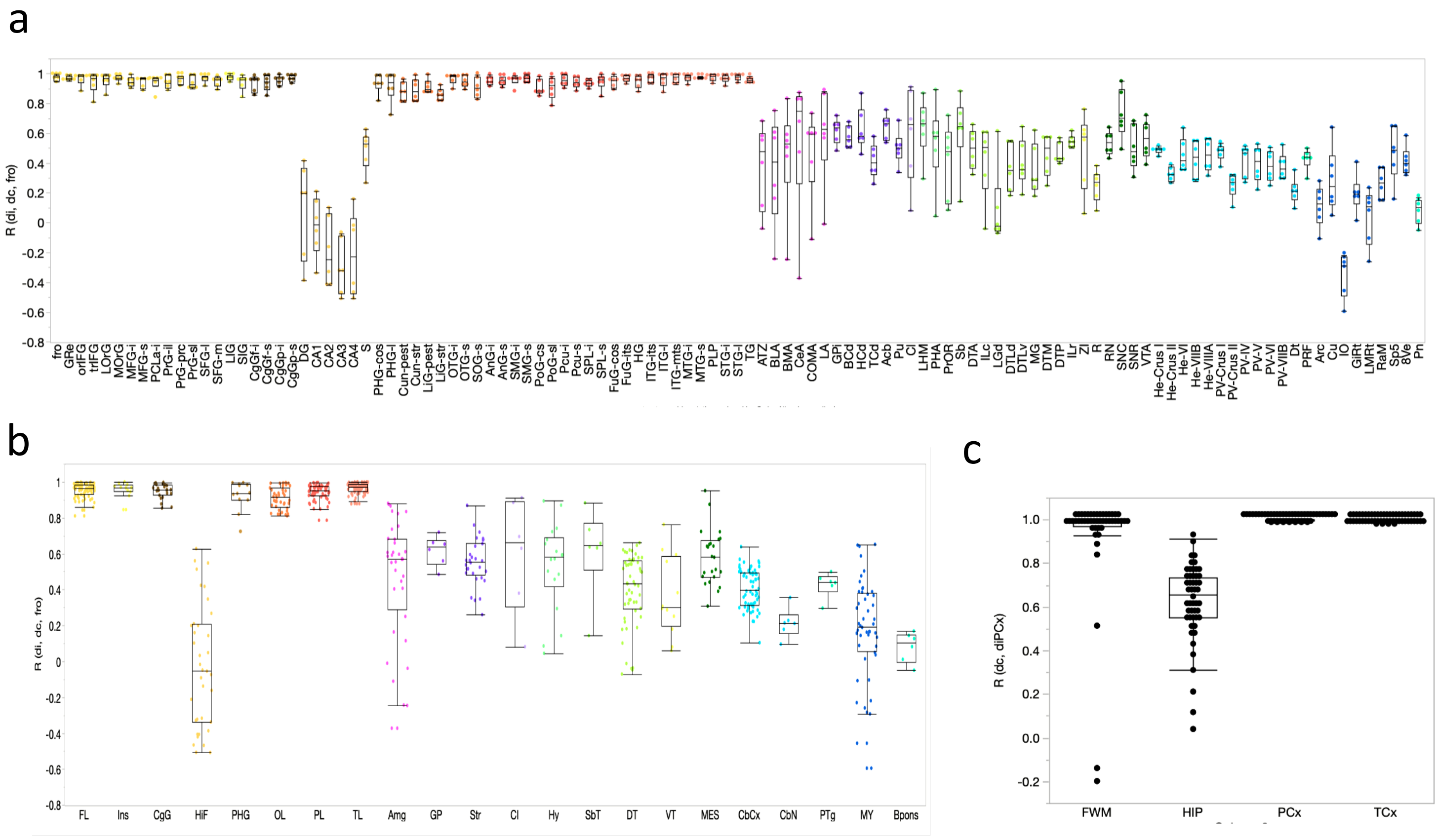

Supplementary Fig. 6. Inter-individual variability across brain regions. a, Euclidian distances of AMPARs subunits per structure (111) per subject (n = 6; d_i) were correlated against a standard (frontal operculum, *fro*; d_c) using the microarray dataset without age correction. Structures are organized in a rostro-caudal order. Correlation coefficients (R) closer to one indicates higher similarity in expression patterns between the structures and the standard. Each dot is a single subject containing the collective information of the four AMPARs subunits. **b,** Same information as in a grouped by major structure. Please see Supplementary Data 2 for definition of abbreviations. **c,** Euclidian distances of AMPARs subunits per structures (4) and per subjects (n = 50) were correlated against the Parietal Cortex, PCx using ADTBI dataset not corrected by age. The hippocampus (HIP) is drastically different from the other three regions and also highly variable between subjects. FWM, forebrain white matter, TCx, Temporal cortex. Each dot is a single subject.

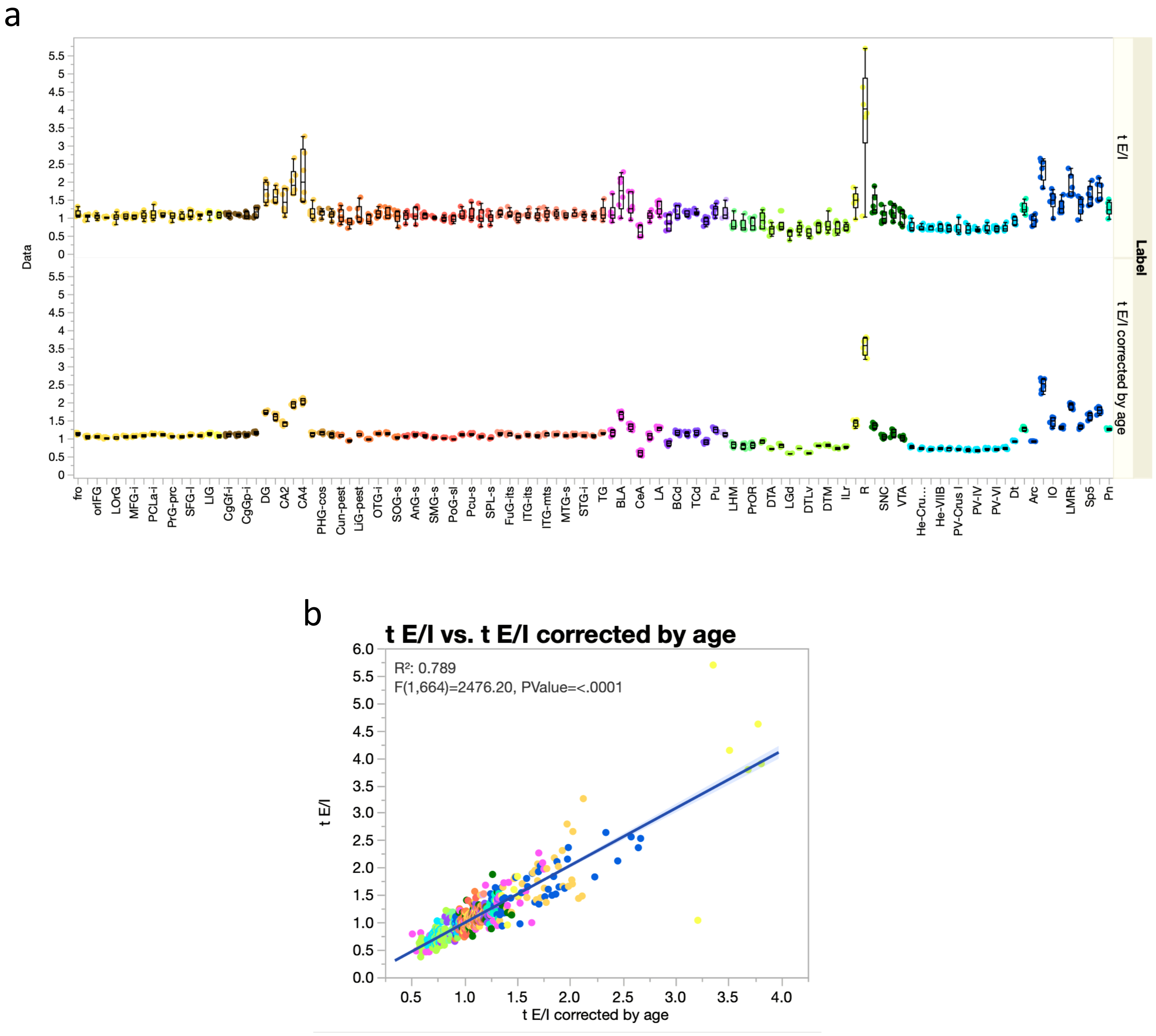

Supplementary Fig. 7. Structural differences in transcriptional E/I ratio in the human brain. a, Box plots of *t*E/I (total expression of AMPARs subunits over total expression of $GABA_A$Rs subunits) across the whole human brain. The ratio before (top) and after age correction (bottom) were calculated for each region and were not different within brain structures. Please see Supplementary Data 7 for statistical analysis. Structures were ordered along the anteroposterior axis. Each dot represents a single subject. **b,** Pearson correlation between *t*E/I corrected and not corrected by age.

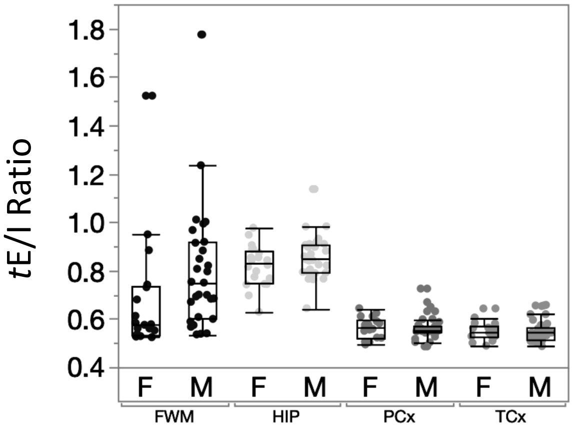

Supplementary Fig. 8. No significant effect of sex on the tE/I ratio. Box plots, described as in supplementary figure 1, showing the *t*E/I ratio grouped by sex in the forebrain white mater (FWM), hippocampal formation (HIP), the parietal and tempral cortices (PCx and TCx) available in the ADTBI dataset. No differences were observed by double tailed student *t* test. *P* = 0.11 (n = 18 F and 29 M), *P* = 0.15 (n = 20 F and 30 M), *P* = 0.90 (n = 18 F and 28 M) and *P* = 0.89 (n = 19 F and 31 M) for FWM, HIP, PCx and TCx respectively. Each dot represents a single subject.

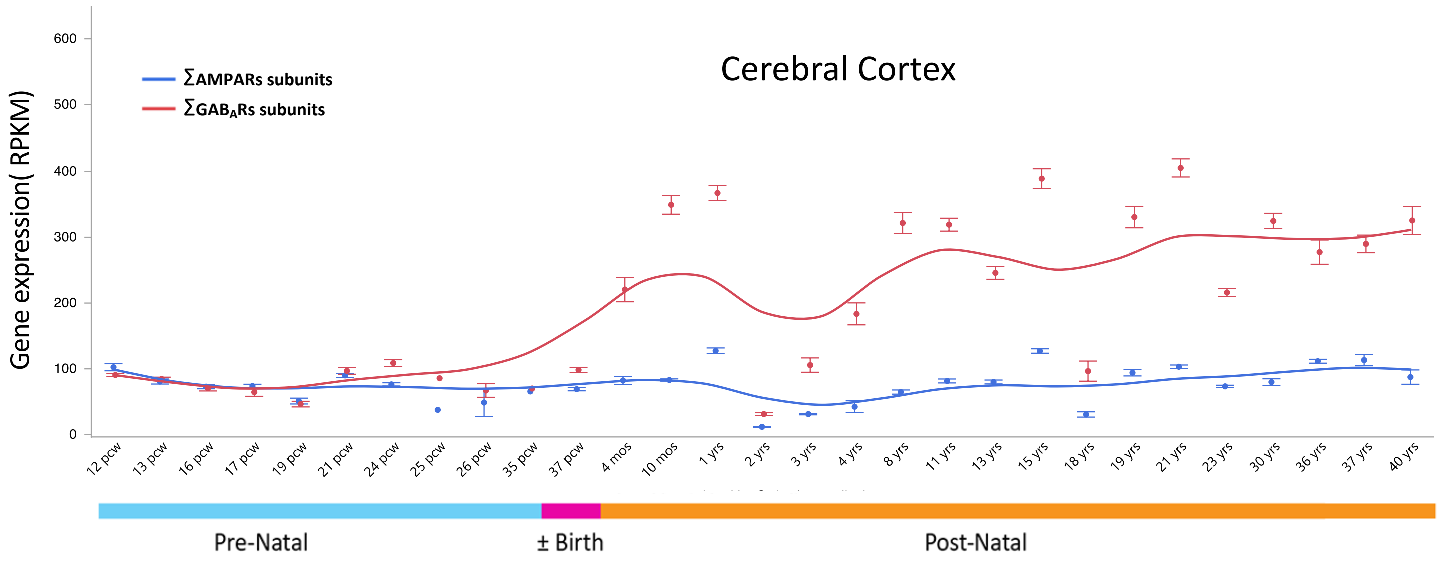

Supplementary Fig. 9. ΣAMPAR and ΣGABA$_A$R subunits during the development in cerebral cortex. Y-axis displays mRNA expression levels (Log2). X-axis shows subject age, as categorical values, from 12 post-conception weeks (pcw) to 40 years according to the Brainspan dataset. Data are represented as mean ± SEM. Spline smooth curves show the principal trend in values for AMPA and GABA$_A$ receptors.

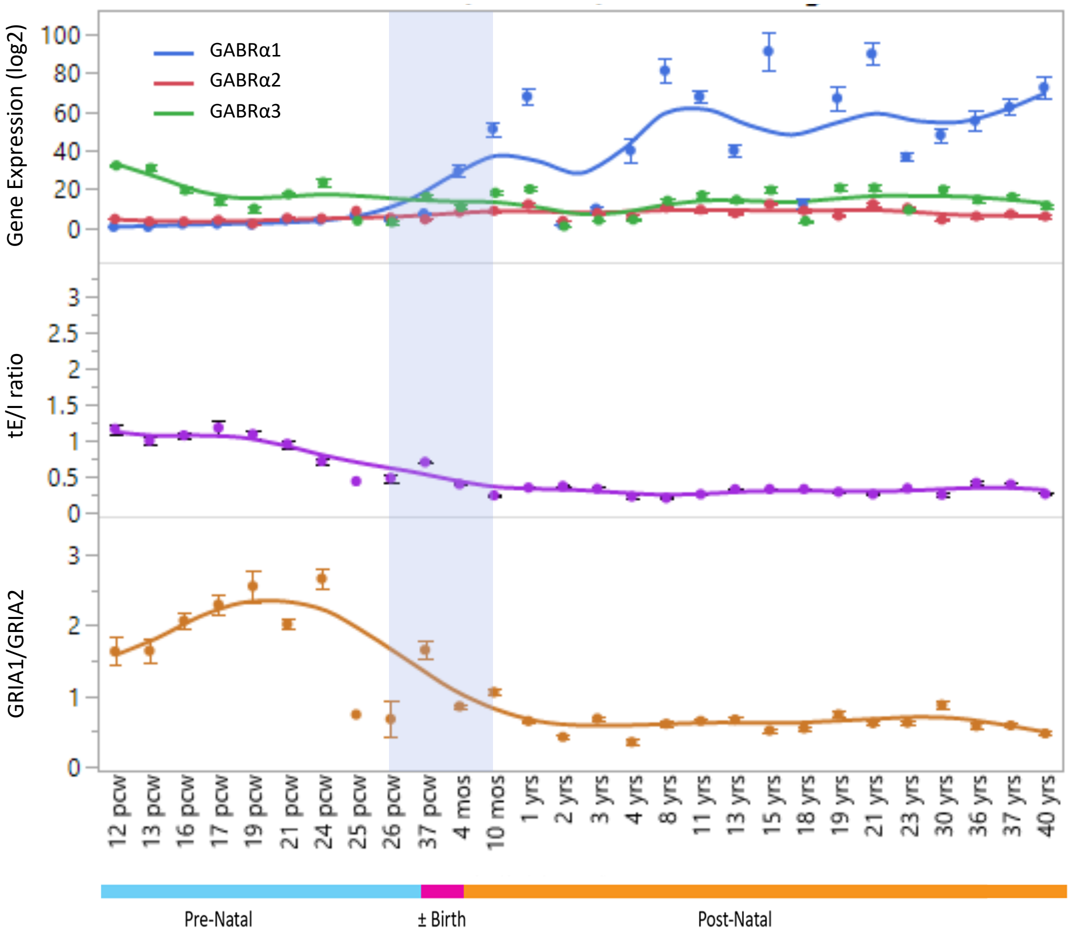

Supplementary Fig. 10. Gene expression for selected GABA$_A$Rs and AMPARs and their relationship with the *t*E/I ratio during development. Y-axes display mRNA expression levels (log2). X-axis shows subject age from 12 pcw to 40 years, as categorical values, according to Brainspan dataset. Data are expressed as mean ± SEM. Spline smooth curves show the principal trend in values for gene expression values
